# Structures of *apo* Cas12a and its complex with crRNA and DNA reveal the dynamics of ternary complex formation and target DNA cleavage

**DOI:** 10.1371/journal.pbio.3002023

**Published:** 2023-03-14

**Authors:** Li Jianwei, Chacko Jobichen, Satoru Machida, Sun Meng, Randy J. Read, Chen Hongying, Shi Jian, Yuren Adam Yuan, J. Sivaraman

**Affiliations:** 1 Department of Biological Sciences, National University of Singapore, Singapore, Singapore; 2 Department of Haematology, University of Cambridge, Cambridge, United Kingdom; ETH Zurich, SWITZERLAND

## Abstract

Cas12a is a programmable nuclease for adaptive immunity against invading nucleic acids in CRISPR–Cas systems. Here, we report the crystal structures of apo Cas12a from *Lachnospiraceae bacterium MA2020 (Lb2)* and the *Lb2*Cas12a+crRNA complex, as well as the cryo-EM structure and functional studies of the *Lb2*Cas12a+crRNA+DNA complex. We demonstrate that *apo Lb2*Cas12a assumes a unique, elongated conformation, whereas the *Lb2*Cas12a+crRNA binary complex exhibits a compact conformation that subsequently rearranges to a semi-open conformation in the *Lb2*Cas12a+crRNA+DNA ternary complex. Notably, in solution, *apo Lb2*Cas12a is dynamic and can exist in both elongated and compact forms. Residues from Met493 to Leu523 of the WED domain undergo major conformational changes to facilitate the required structural rearrangements. The REC lobe of *Lb2*Cas12a rotates 103° concomitant with rearrangement of the hinge region close to the WED and RuvC II domains to position the RNA–DNA duplex near the catalytic site. Our findings provide insight into crRNA recognition and the mechanism of target DNA cleavage.

## Abstract 2

CRISPR‒Cas is an adaptive immune system against invading nucleic acids. In this study, the structures of apo Cas12a, Cas12a-crRNA and Cas12a-crRNA-DNA reveal Cas12a’s unique elongated form, its mechanism of crRNA binding and the conformational dynamics required to target its substrate DNA for cleavage.

## Introduction

The CRISPR–Cas (clustered regularly interspaced short palindromic repeats/CRISPR-associated protein) system is a bacterial adaptive immune system against invading nucleic acids [1,2]. CRISPR–Cas systems have been extensively studied over the past decade [3–10], and knowledge of their unique activity has paved the way for the development of next-generation, high-throughput genome editing tools [5,11,12]. Among the various Cas enzymes, the type V-A effector protein, Cas12a, previously termed Cpf1 [3,13,14], is known to process precursor-crRNA into mature crRNA without the requirement of transactivating RNA, a small trans-encoded RNA, which mediates the maturation of crRNA in CRISPR–Cas9. Instead, Cas12a targets the invading DNA through RNA–DNA base-pairing [14,15]. Cas12a recognizes a 5′ TTTN- protospacer adjacent motif (PAM) [3,16,17] and produces staggered ends at a position 18 to 20 bp downstream of PAM [3]; this differs significantly from the well-characterized *S*. *pyogenes* Cas9, which recognizes a 3′ NGG- PAM [3,16–18] in target double-stranded DNA (dsDNA) and produces blunt ends at a position 3 bp upstream of the PAM. The double-strand breaks in dsDNA are joined by nonhomologous end-joining (NHEJ) repair. In CRISPR–Cas12a, a PAM that is 18 nt from the double-strand cleavage site provides the possibility for secondary cleavage after NHEJ repair. After cleaving the target dsDNA, Cas12a acquires indiscriminate ssDNase activity, referred to as trans-cleavage activity [17,19,20]. This property allows Cas12a to be used to detect trace amounts of DNA, for example, trace amounts of viral genome in DNA Endonuclease Targeted CRISPR Trans Reporter (DETECTR) assays [17,19–21]. Thus, CRISPR–Cas12a is an attractive alternative strategy for next-generation genome editing and diagnostics.

Several Cas12a orthologs have been utilized for genome editing to date [14,22,23]. These orthologs share various structural features, as identified through X-ray crystallography and Cryo-EM structural analysis of crRNA-bound and crRNA-DNA complexes of the Cas enzyme bound to crRNA and DNA fragments. Typically, Cas12a exhibits a bilobed architecture: a recognition lobe (the REC1 and REC2 domains), and a nuclease (NUC) lobe (the WED, PI, RuvC, BH, and Nuc domains) for cleaving nucleic acids [24–26]. The crRNA pseudoknot structure is anchored in the WED domain and extensively interacts with the RuvC and REC2 domains [24,25]. The 3′ tail of the crRNA hybridizes to the target DNA strand, forming an R-loop [26], which is accompanied by interactions with the REC and NUC lobes. A molecular dynamics study of *Francisella novicida* Cas12a indicated flexibility of the PI domain when in complex with crRNA and rigidity when the PI domain is engaged as part of the Cas12a+crRNA+DNA ternary complex [27]. Single-molecule fluorescence resonance energy transfer (FRET) studies of *Fn*Cas12a and *Lachnospiraceae bacterium ND2006* (*Lb*) Cas12a further demonstrate that Cas12a adopts a compact shape in the crRNA-bound conformation [28,29], with the lobes undergoing slight opening upon DNA binding.

Curiously, FRET studies indicate that *apo* Cas12a has an additional elongated conformation distinct from the semiclosed conformation of Cas12a+crRNA+DNA [28,29]. These studies collectively suggest the presence of multiple conformations in equilibrium, with respective domain flexibility differing in each conformation [28,29]. Indeed, conformational equilibrium is necessary for the activity of Cas12a, as suggested by the activity of the AcrVA4 inhibitor, which suppresses the activity of *Lb*Cas12a by making the RNA-bound compact structure rigid [30]. High structural plasticity is implicated in the mechanism of crRNA capture and substrate binding, and yet, despite the wealth of knowledge available, the structural transitions from *apo* Cas12a to the RNA/DNA-bound forms are not fully understood.

Among the Cas12a orthologs, *Lachnospiraceae bacterium MA2020 (Lb2)* Cas12a is the smallest in molecular weight and recognizes a short spacer (14 nt) for cleavage and the creation of indels with high fidelity [23]. Here, we report the crystal structures of *apo Lb2*Cas12a and the *Lb2*Cas12a+crRNA binary complex as well as the cryo-EM structure of the *Lb2*Cas12a+crRNA+DNA ternary complex. These structures reveal distinct conformations of *Lb2*Cas12a at each interaction stage. Furthermore, through functional studies, we identified the mechanism of crRNA binding and targeted DNA cleavage.

## Results

### The TTNN PAM sequence is recognized by *Lb2*Cas12a

We first explored the mechanism of crRNA-dependent dsDNA cleavage by *Lb2*Cas12a. In the presence of Mg^2+^, wild-type *Lb2*Cas12a (MW, 141 kDa) exhibits strong catalytic activity against the dsDNA substrate; 20 nt crRNA was used as the guide strand (S1A–S1C Fig). Sequencing analysis revealed the production of staggered ends downstream of PAM at nucleotides 14–17 in the nontargeted (noncomplementary) strand and at nucleotide 23 in the targeted (complementary) strand (S1B Fig). The breakage sites of the nontarget strand are consistent and observed in its orthologs [31], which may be due to the repetitive endonucleolytic cleavage of the nontarget strand. The targeted strand forms a heteroduplex with crRNA and is stabilized by the REC lobe. We determined that *Lb2*Cas12a recognizes and cleaves target dsDNA at the 5′-TTNN-3′ PAM (N = A, T, G, or C); this recognition sequence was deemed most effective when compared with other cleavage sequences (S1D Fig). Of note, others have demonstrated that *Acidaminococcus* sp. (*As*)Cas12a and *Lachnospiraceae bacterium ND2006* (*Lb*)Cas12a orthologs do not recognize the 5′-TTTT-3′ PAM [32]. Together, these observations indicate that *Lb2*Cas12a minimally requires dinucleotide PAM.

### Overall structures of *Lb2*Cas12a

**Fig 1:**
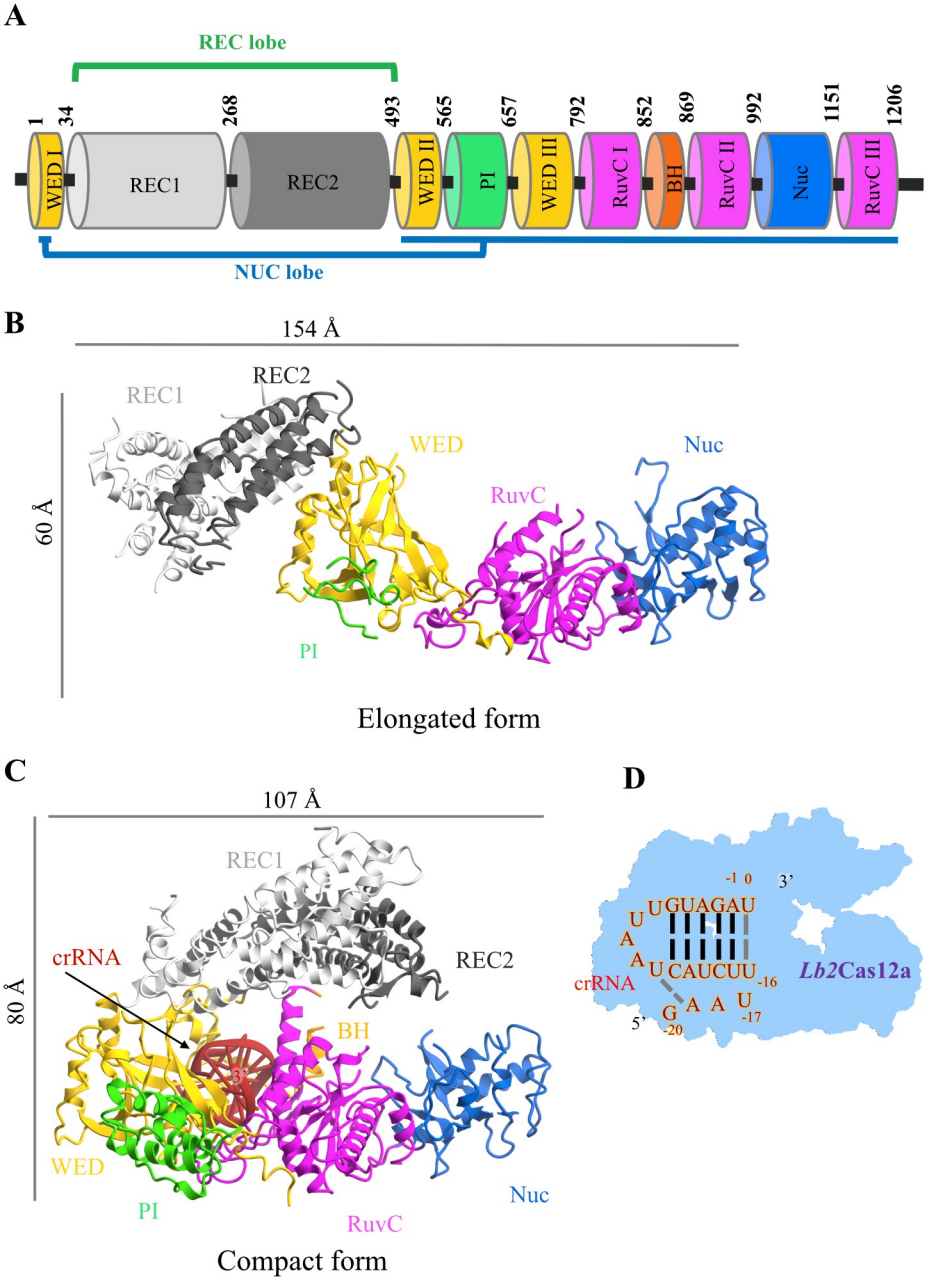
Crystal structures of *Lb2*Cas12a. (A) Bar diagram showing the domain organization of *Lb2*Cas12a. Different domains are distinguished by color. (B) Overall structure of the *apo Lb2*Cas12a, BH domain is disordered in *apo* and not modeled. (C) Overall structure of the *Lb2*Cas12a-crRNA complex. (D) Schematic diagram of the *Lb2*Cas12a-crRNA, black line: Canonical base pairs; gray line: noncanonical base pairs. The structure-related figures in this paper are prepared by CueMol2 program.

The crystal structure of full-length *Lb2*Cas12a (residues 1–1,206 aa) was determined at 3.1-Å resolution (S1 Table) (PDB ID: 8H9D). Strikingly, both *apo* and RNA-bound *Lb2*Cas12a are present in the asymmetric unit of the crystal. Each *Lb2*Cas12a monomer comprises an α-helical recognition lobe (34–492 aa) followed by a nuclease (NUC) lobe (1–33 aa, 493–1,206 aa) (Fig 1A–1C). These 2 lobes are naturally connected by flexible loops (Fig 1B and 1C). The 20 nucleotides of the crRNA in the complex are well defined in the electron density map (Fig 1D and S2A and S2B Fig). Notably, there are differences in the conformations between the *apo* and RNA-bound forms of *Lb2*Cas12a. The 2D class averages for negative staining of *apo Lb2*Cas12a indicated multiple conformations, whereas *Lb2*Cas12a-crRNA persisted with a uniform, compact conformation (S3A Fig), which is similar to the study of orthologs [25] of *Lb2*Cas12a. In addition, after incubation with trypsin, *apo Lb2*Cas12a degraded faster than *Lb2*Cas12a-crRNA, which is indicative of a different conformation between the *apo* and RNA-bound forms (S3B Fig). These results suggest that *apo Lb2*Cas12a exists dynamically between elongated and compact forms and that crystal packing may favor the capture of the elongated form in the asymmetric unit.

### The elongated structure of *apo Lb2*Cas12a

In *apo Lb2*Cas12a, the N-terminal REC1 and REC2 domains (REC lobe) and the C-terminal wedge (WED), RuvC, and Nuc domains (NUC lobe) (Fig 1A) are arranged in an elongated conformation, with the REC1 domain and Nuc domain at each end (Fig 1B). Two flexible loops (Asn33–Asn45 and Lys478–Thr498) connect the REC and NUC lobes. The WED domain connects the REC2 and RuvC domains and forms 2 positively charged cavities within these domains (S4A Fig). Using a DALI structural homolog search [33], we were unable to identify any similar structure for the full-length *apo Lb2*Cas12a in the PDB database (S2 Table). However, structural homologs were identified for both the independent NUC and REC lobes (S3 and S4 Tables). These results indicate that *apo Lb2*Cas12a exhibits a unique, elongated conformation, with distinct spatial arrangement of the domains.

### The *Lb2*Cas12a+crRNA binary complex adopts a compact structure

**Fig 2:**
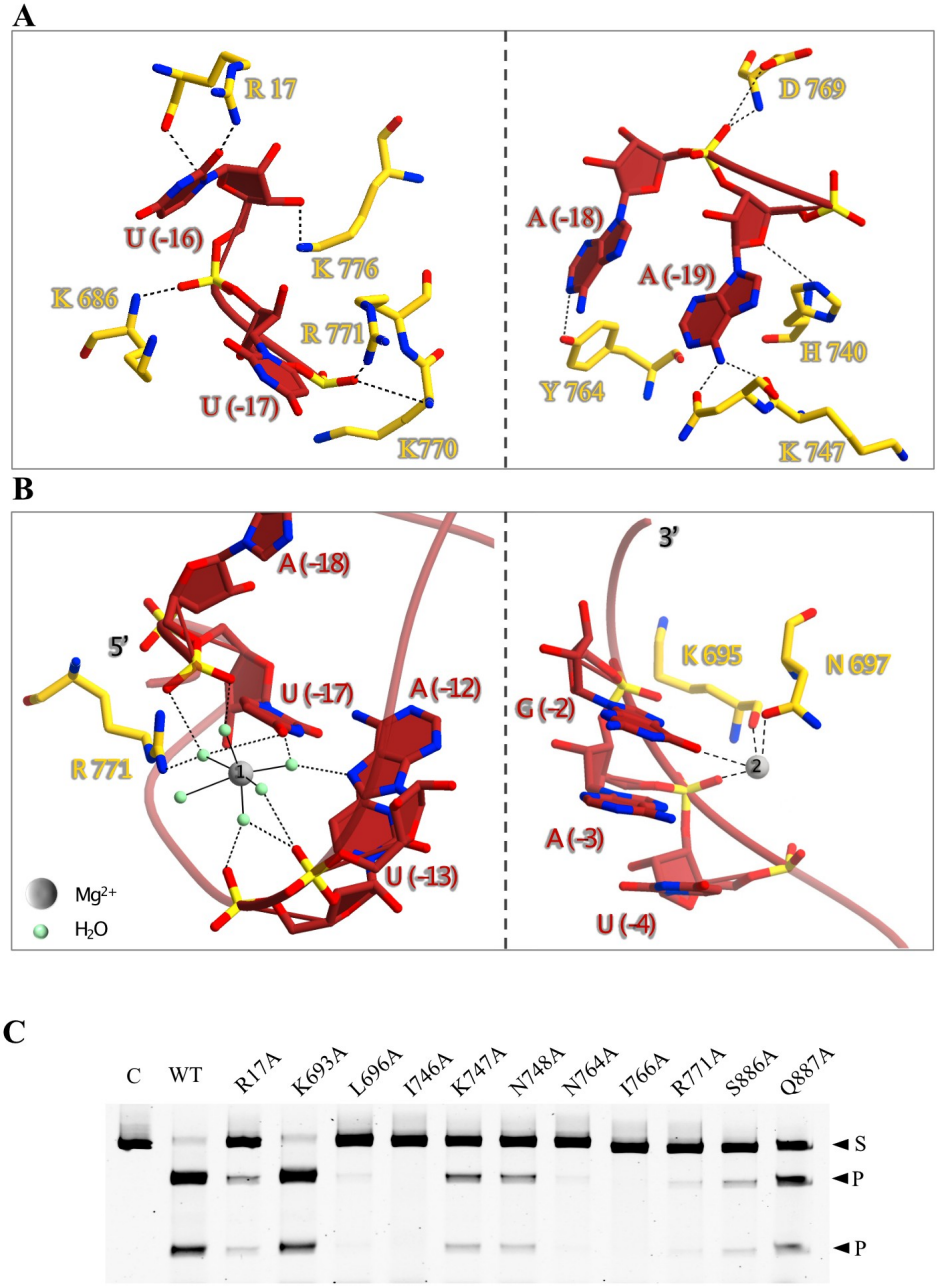
Recognition of the crRNA by *Lb*2Cas12a. (A) Interactions between the 5′ pseudoknot of crRNA and *Lb2*Cas12a residues. (B) Interactions between the hydrated magnesium ions and crRNA, Mg^2+^ and H_2_O are indicated by gray and green, respectively. (C) dsDNA cleavage activity analysis of *Lb2*Cas12a mutants in RNA recognition region. C: Control; only target dsDNA, S: Substrate, P: Cleaved product.

The overall 3D structure of the *Lb2*Cas12a+crRNA binary complex adopts a triangular, compact conformation (Fig 1C). The REC and NUC lobes lie adjacent to each other in 3D space, creating a positively charged cavity in the center (Fig 1C and S4B Fig). Trp871 of the RuvC II domain, lying close to the Bridge Helix (BH) region (Leu477–Lys493), is anchored in the hydrophobic pocket of the REC2 domain (S5A Fig). The first helix (Trp871–Tyr899) of the RuvC II domain is analogous to the suspension helix of orthologous protein structures that forms a loop upon binding to DNA [29,34]. Lys869, located between the BH and RuvC II domains, forms electrostatic interactions with the REC2 domain. These interactions stabilize the compact conformation, as reported elsewhere for orthologous proteins [35,36] (S5 Table). crRNA is anchored in the positively charged cavity and forms the pseudoknot of the 5′ handle through intramolecular base-pairing; this interaction is coordinated by hydrated magnesium ions and highly conserved WED domain residues (Fig 2A and 2B and S2B Fig). Indeed, alanine mutations at these conserved sites (Arg17, Leu696, Ile766, and Ile746) nearly completely abolish dsDNA cleavage activity (Fig 2C and S2C Fig). These observations indicate that conserved WED domains coordinate the interactions of the REC and NUC lobes with crRNA to maintain a compact conformation.

### Comparison of *apo Lb*2Cas12a and *Lb2*Cas12a+crRNA binary complex structures

**Fig 3:**
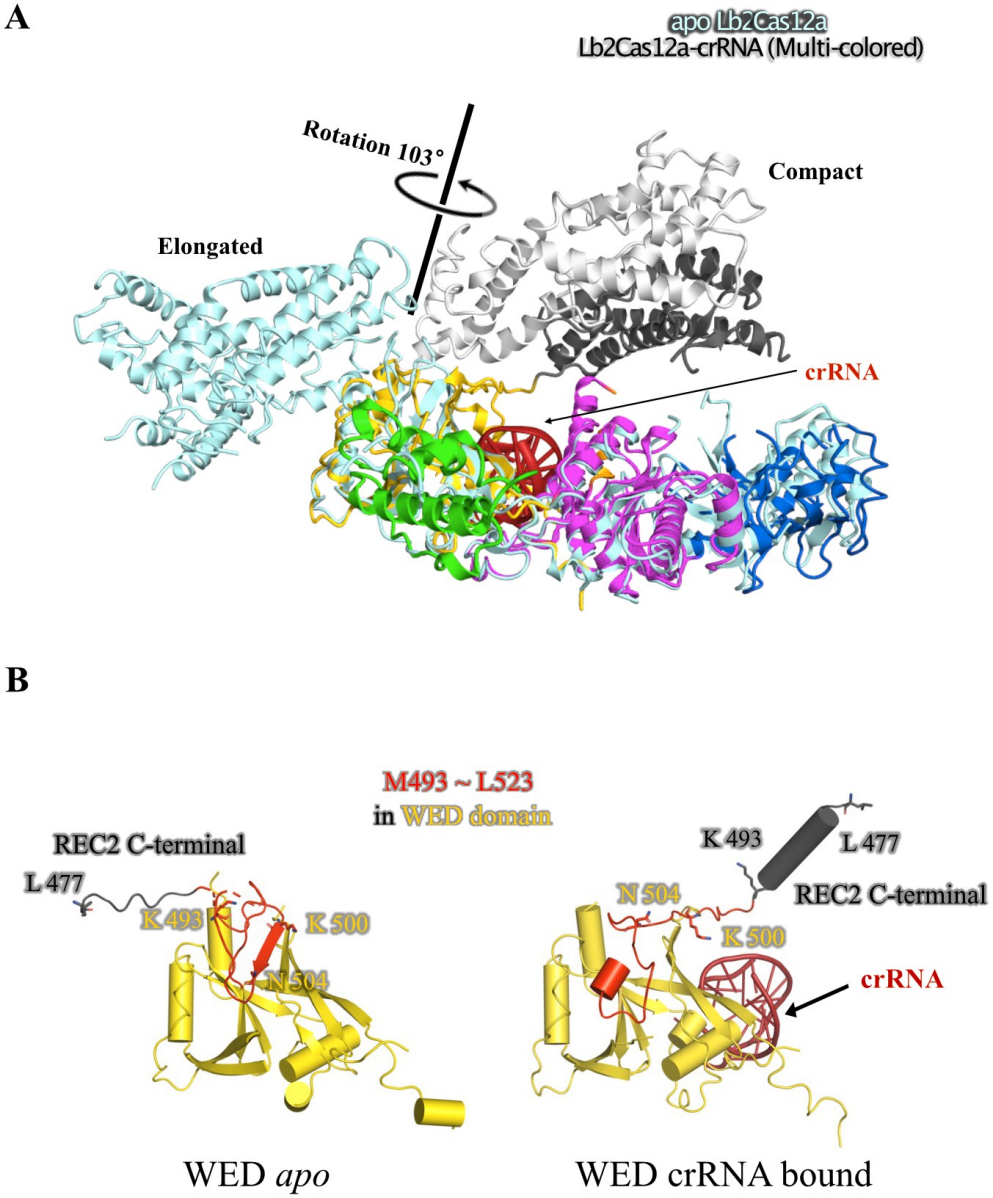
Structural rearrangement of *Lb2*Cas12a is triggered by binding crRNA. (A) Superposition of NUC lobe of *apo Lb2*Cas12a and *Lb2*Cas12a-crRNA, structure of *apo* and RNA bound *Lb2*Cas12a represented as ribbon diagram, Asp200 and Gly524 in *apo* and RNA bound form shown as sticks. (B) N-terminus of RuvC domain in *apo* and RNA bound form, helices represented by cylinder, key residues shown as sticks.

Superposition of the *apo Lb2*Cas12a and *Lb2*Cas12a+crRNA binary complex structures demonstrates a 103° rotation of the REC lobe with respect to the α-helix of the WED-I domains of the crRNA complex in its compact conformation (Fig 3A). Residues from the region Met493–Leu523 of the WED domain participate in major conformational changes to facilitate the structural rearrangements associated with this rotation (Fig 3B, S1 Video). The structural comparison further demonstrates a conformational rearrangement of the linker region between the REC2 and RuvC domains, although there were no structural changes observed within the domains (S6 Table). The flexible loop (Leu477–Lys493) connecting the REC and RuvC domains of *apo Lb2*Cas12a transforms into an α-helix in the compact conformation, folding back at the C-terminus of the REC2 domain. Meanwhile, in this compact conformation, a β-strand (Lys500–Asn504) in the *apo Lb2*Cas12a becomes a short loop (Lys500–Asn504) (Fig 3B), a change that is likely supported by the interaction of residue Lys500 with the crRNA backbone. Significantly, the loop-turned-α-helix (Leu477–Lys493) harbors a hydrophobic patch that interacts with Trp871, linking the REC and NUC lobes through the BH and RuvC II suspension helix (S5A Fig). Without crRNA, residues Tyr484, Leu491, and Thr492 in the REC2 domain remain separated and do not interact with Trp871. PISA (Protein Interfaces, Surfaces and Assemblies) analysis indicates that the *Lb2*Cas12a+RNA complex gains a buried area of 2,436 Å^2^ compared with *apo Lb2*Cas12a. Collectively, crRNA binding and rearrangement of the hinge loop precedes the formation of the PAM-binding channel.

### crRNA binding

Next, we analyzed the extensively positively charged RNA-binding pocket (S4A Fig) of *Lb2*Cas12a, anticipating that the positive charges in this region (Lys686–His699, Gly883–Asn900) play a role in maintaining its elongated conformation (S5B Fig). Upon interacting with crRNA, the positively charged pocket within the NUC lobe wraps around the crRNA. Subsequently, the bound crRNA backbone alters the surface charges of the pocket. These interactions of the REC lobe with crRNA result in a compact triangular conformation (Fig 1C and S4B Fig) that is stabilized by a hydrophobic cluster among Trp871, Tyr484, Leu491, and Thr492 and several hydrogen-bonding contacts through residues Asp39, Tyr149, Asn504, Glu875, and Thr492 (S5A Fig).

Next, to understand the role of electrostatic surface potential changes caused by the binding of crRNA, we assessed the role of pH. Through gel filtration and dynamic light scattering (DLS) analyses, we noted that the overall size of *apo Lb2*Cas12a depends on the pH of the solution (S6A and S7A–S7C Figs); for example, at pH 7.4, the *Lb2*Cas12a+crRNA complex is smaller than *apo Lb2*Cas12a (S6B and S7B and S7D Figs). To further assess the involvement of surface charge in these size discrepancies, we mutated 4 basic residues (Arg864Glu-Lys866Glu-Arg868Glu-Lys869Glu) of the BH domain at the ridge of the positively charged cavities. This quadruple mutant was unable to bind crRNA; this observation is similar to that with the PI domain-deletion mutant (ΔPhe558~Thr660) (S8 Fig). However, through DLS (S7F and S7G Fig) and gel filtration (S6C Fig), we identified that the size of the quadruple mutant remained unchanged by the presence or absence of crRNA. Collectively, these observations indicate that the electrostatic environment and RNA binding contribute to the observed conformational rearrangement.

### RNA–DNA complex formation drives rearrangements of the dynamic REC lobe

**Fig 4:**
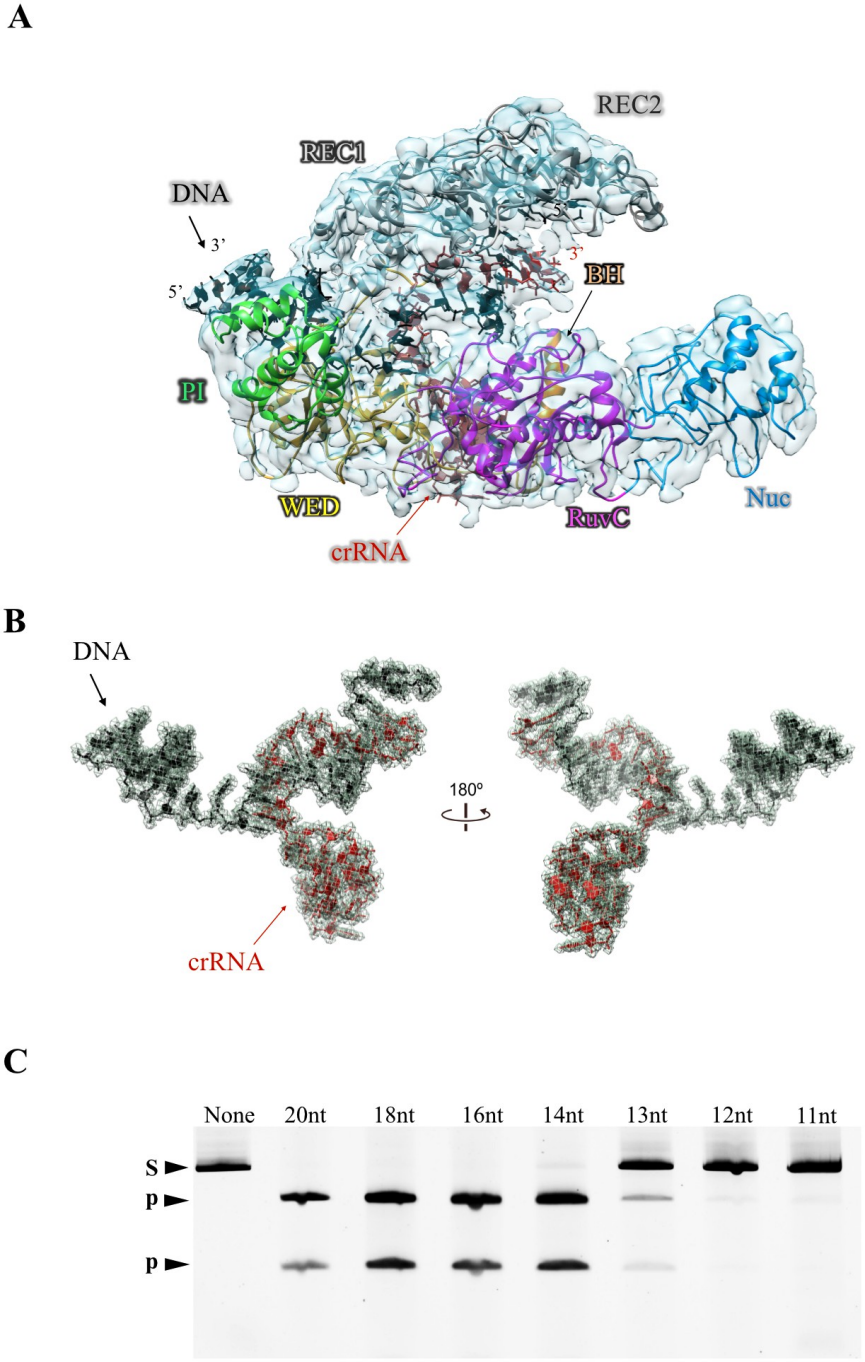
Cryo-EM structures of *Lb2*Cas12a-crRNA-DNA complex. (A) Overall structure of the *Lb2*Cas12a-crRNA-DNA, at 3.95 Å resolution. These figures are prepared by Chimera program; different domains are distinguished by color, surface transparency: 70%. (B) crRNA-DNA duplex in Cryo-EM density map. (C) The guide strand length of crRNA required for *Lb2*Cas12a to achieve cleavage activity. None: only target dsDNA, S: target dsDNA substrate, P: cleavage product.

Next, we determined the cryo-EM structure of the *Lb2*Cas12a+crRNA+DNA ternary complex at 3.95 Å resolution (Fig 4A, S7 Table) (PDB ID 8I54). This structure is reminiscent of the conformation of Cas12a+crRNA+DNA ternary complexes reported for other Cas12a orthologs (Fig 4, S2 Table). Of note, the ternary complex contains an RNA–DNA heteroduplex of only 14 bp that mimics the cleaved heteroduplex that would exist in situ (Fig 4B); this 14-bp heteroduplex is shorter than the 20-bp heteroduplex used previously to study Cas12a orthologs in target cleavage conformation assays [26,28,34,37].

In our Cas12a+crRNA+DNA ternary complex structure, the REC domain is rotated compared with the binary complex (S9A Fig), forcing the REC linker (261Leu–273Ser) toward the 14-bp heteroduplex. The RNA–DNA duplex passes through a central channel formed by the REC and NUC lobes, which results in the expansion of the central channel (S4C Fig). Unlike *Fn*Cas12a [28,29], the REC and NUC lobes in *Lb2*Cas12a are not connected through a Nuc domain stem (Arg1056–Asn1066), which permits flexibility between these lobes in *Lb2*Cas12a. The 14-bp heteroduplex is not sufficiently long to stabilize the DNA-REC2 contact through the finger helix (234Lys~244Gln) [24,28]. Consistently, the 14-nt guide strand of crRNA is the minimum length for efficient cleavage of target dsDNA by *Lb2*Cas12a (S4C Fig). Finally, in the ternary complex, the PI domain is rotated by 38° and lies closer to the PAM nucleotides of the dsDNA (S10A Fig). Based on these structures, we hypothesize that residues Lys575 and Lys518 of *Lb2*Cas12a play a role in PAM recognition (S10B Fig). Consistently, mutation of Lys575 or Lys518 weakens or abolishes dsDNA cleavage activity (S10C Fig). Rearrangement of the hinge region is also observed in the transition from the binary to the ternary complex.

Further comparison confirms that the REC lobe is highly flexible (S9B Fig), which is consistent with its orthologs [28]. This finding suggests that there is movement of the domains during heteroduplex formation. These conformational changes are consistent with the DLS and gel filtration results, in which the ternary complex exhibits a slightly larger size than the binary complex at a constant pH of 7.4 (S6B and S7D and S7E Figs). Thus, our comparison of the pre-cleavage binary complex with the post-cleavage ternary complex reveals that bilobed movement drives the structural rearrangement of the ternary complex.

### The mechanism of DNA cleavage

The *Lb2*Cas12a catalytic site for DNA cleavage is within the RuvC domain, which is formed by 3 conserved residues: Asp815, Glu906, and Asp1161 (S13A and S14 Figs). We demonstrated that alanine substitution of these 3 residues abolished dsDNA cleavage activity (S13B Fig). In addition, alanine substitution of the conserved catalytic residue Arg1124 in the Nuc domain significantly weakened dsDNA cleavage activity (S13B Fig), suggesting that the Nuc domain assists with double strand cleavage by the RuvC domain. After targeted cleavage of dsDNA and formation of the RNA–DNA duplex, *Lb2*Cas12a then initiates trans-cleavage activity (S13C Fig). Consistently, the “Lid” structure (S13A Fig), which mediates trans-cleavage activity in previously reported ortholog studies [28], was also observed in *Lb2*Cas12a, indicating that the Cas12a family of proteins adopts a similar mechanism to activate trans-cleavage activity. Notably, the presence of Mn^2+^ triggers indiscriminate ssDNA cleavage activity of *apo Lb2*Cas12a (S13C Fig), which is consistent with previous studies [38]. Overall, we demonstrate that the RuvC catalytic residues Asp815, Glu906, and Asp1161, along with Arg1124 of the Nuc domain (S13C Fig), are responsible for the trans-cleavage activity of *Lb2*Cas12a, which is consistent with a previous study in which the Nuc domain assists the RuvC domain in achieving trans-cleavage [35].

## Discussion

In this study, we report the crystal structures of *apo Lb2*Cas12a and the *Lb2*Cas12a+crRNA binary complex, along with the cryo-EM structure of the *Lb2*Cas12a+crRNA+DNA ternary complex. Previous negative-staining EM [25] and SAXS (small-angle X-ray scattering) data [39] suggest that *apo*Cas12a orthologs adopt an elongated shape. Our crystal structure reveals that *Apo Lb2*Cas12a exhibits a unique, elongated structure, which is supported by DLS and gel filtration chromatography profiles. Furthermore, we demonstrate that the *Lb2*Cas12a+crRNA binary complex adopts a compact triangular structure, which is consistent with previous findings in orthologs [25,35]. We demonstrate that structural rearrangement of the hinge loop into an α-helix (Leu477–Lys493) leads to the formation of a hydrophobic cluster among Trp871, Tyr484, Leu491, and Thr492. Meanwhile, several hydrogen bonds are formed between Asp39, Tyr149, and Asn504 and between Glu875 and Thr492, with this compact conformation further stabilized by hydrophobic interactions between the newly formed α-helix and the conserved Trp871 located between the BH and RuvC II domains. Thus, our findings indicate that the formation of the α-helix (Leu477–Lysr493)—induced by crRNA—is a prerequisite for the formation of the PAM-binding channel.

**Fig 5:**
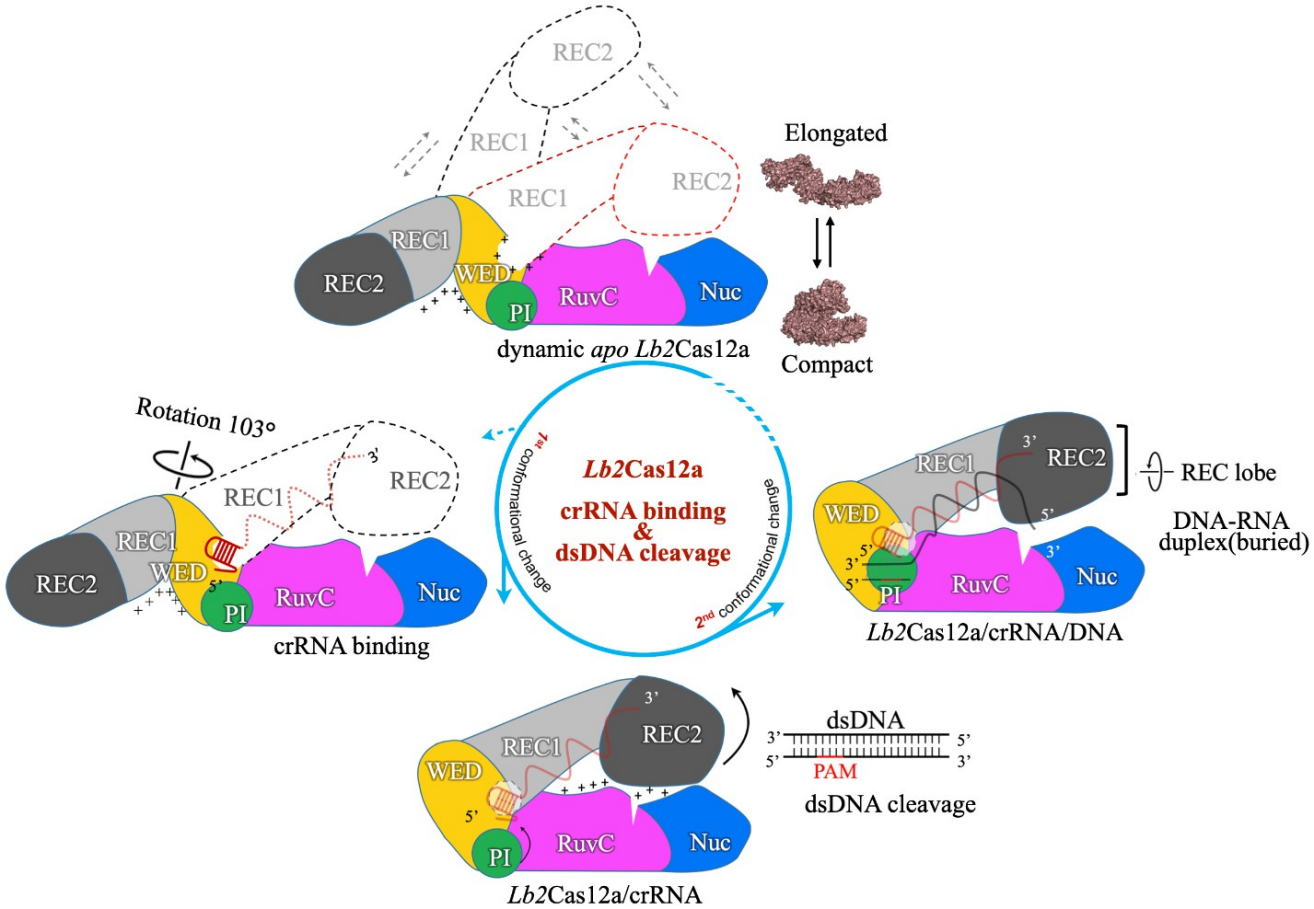
Model of crRNA binding and DNA cleavage triggering conformational changes in *Lb2*Cas12a. A*po Lb2*Cas12a adopts dynamic conformation. The negatively charged crRNA is captured by the positively charged RNA-binding pocket and triggers the REC lobe to rotate to the NUC lobe side to complete the first conformational rearrangement. The PAM sequence on dsDNA is recognized by PI domain and forms a heteroduplex with crRNA to trigger the second conformational rearrangement of REC lobe. The catalytic sites in RuvC and Nuc domain are marked by a triangular gap, and the positively charged RNA-binding sites are indicated by a polygonal split. Different domains are distinguished by color.

The 2D class averages of negative staining images from our study suggested that *apo Lb2*Cas12a adopts a variety of conformations, including both elongated-open and compact-closed conformations, whereas *Lb2*Cas12a-crRNA adopts a uniform compact conformation. These findings indicate that the 2 lobes in *apoLb2Cas12a* are dynamic. We propose that the dynamic conformation is physiologically relevant for 2 reasons. First, it exposes the peptide bonds of the flexible linkers for hydrolysis. Indeed, *apo Lb2*Cas12a was more susceptible to degradation by trypsin than *Lb2*Cas12a+crRNA. Hence, we speculate that the elongated conformation of *apo Lb2*Cas12a improves its rate of elimination from the cell without mounting an unnecessary immune response. Second, the dynamic conformation favors the binding of crRNA, with basic residues exposed to the solvent. Notably, the DLS and gel-filtration chromatography results both suggest that the diameter of *apo Lb2*Cas12a varies with respect to the buffer pH. We propose that the altered surface charge that occurs in response to this varied environment triggers the opening and closing of the lobes (Fig 5). In the process of crRNA binding, the negatively charged crRNA backbone is anchored into the RNA-binding pocket through electrostatic interactions. The positively charged cavity formed by the REC lobe and the WED domain is attracted to the crRNA, and these regions are then drawn closer to the NUC lobe. The N-terminal flexible loop of the WED domain allows for rotation and translation of the REC lobe to occur commensurate with the electrostatic forces. The basic residues located in the BH region between Arg864 and Lys870 then interact with the REC2 domain to stabilize this new RNA-bound conformation.

In the transition to a ternary complex, mutation of the conserved residues responsible for DNA binding of the PI domain (Pro567Ala, Lys580Ala, and Lys581Ala) was not sufficient to inactivate the complex, whereas deletion of the entire PI domain completely blocked dsDNA cleavage activity (S10C Fig). Consistently, the entire PI domain, which is connected by 2 flexible loops, rotates 38° to recognize the PAM sequence. Subsequently, the DNA unwinds and forms an R-loop structure with crRNA, which results in expansion of the channel formed by the REC and NUC lobes. The cleaved target DNA is insufficiently long to stabilize the DNA-REC lobe contact through the finger helix (234Lys–244Gln), which leads to further expansion of the channel and its subsequent preparation for trans-cleavage.

PAM is recognized by the conserved PI domain through Lys575 and Lys518. These residues are conserved in the Cas12a family. Modification of these residues may allow *Lb2*Cas12a to recognize noncanonical PAM sequences [32]. The unique feature of *Lb2*Cas12a in recognizing TTNN PAM sequences is an advantage, as it provides the opportunity to target more genes using a crRNA array.

In summary, in reporting the crystal structure of *apo Lb2*Cas12a and the *Lb2*Cas12a+crRNA binary complex, we have identified the conformational rearrangements and mode of crRNA binding. We demonstrate that the crRNA-bound, compact form of *Lb2*Cas12a is achieved through a unique 103° rotation of the REC lobe and through further interaction between the REC and NUC lobes to maintain and stabilize this compact conformation. This evidence demonstrates that crRNA binding and rearrangement of the hinge loop precedes the formation of a PAM-binding channel. Finally, the *Lb2*Cas12a+crRNA+DNA ternary complex structure reveals bilobed movement and rearrangement of the hinge region, including the PI and WED domains and the connective loops, demonstrating that PAM is recognized by the conserved Lys575 and Lys518 residues and that the RNA–DNA duplex is formed with a minimum of 14 bp. Overall, this study offers snapshots of the catalytic activation process, beginning with the RNA-free elongated conformation and crRNA-bound compact-closed conformation resulting in the DNA-bound, post-cleavage conformation.

## Materials and methods

### *Lb2*Cas12a expression and purification

*Lb2*Cas12a gene was purchased from Addgene and reconstituted in the pET28b vector. *Lb2*Cas12a or mutants with pET 28b-N-6×His tag was heterologously expressed in *BL21(DE3)* pLysS *E*. *coli* bacteria. The engineered bacteria in LB medium were placed in a shaker incubator at 37°C, 220 rpm until it reached an OD600 = 0.6, then added IPTG to a final concentration of 0.4 mM and cultured for 16 h at 16°C, 220 rpm. The harvested bacteria were resuspended in lysis buffer (1 mM DTT, 25 mM KH_2_PO_4_ (pH7.0), 10% glycerol, 500 mM NaCl, 25 mM Tris (pH 7.4), 1 mM EDTA). The high-pressure homogenizer (Avestin) with a cooling system was used for the lysis. The lysed bacteria were centrifuged at 40,000 rpm, 4°C for 1 h by Beckman Ultracentrifuge Type 45Ti. The supernatant was incubated with RNase A at 16°C for 15 min to remove endogenous RNA. Subsequently, the harvested supernatant was purified using Ni^2+^ affinity chromatography column (GE Healthcare) combined with ÄKTA purification system (GE Healthcare). Further purification was carried out by size-exclusion chromatography using the HiLoad Superdex 200 26/60 prep column (GE Healthcare). The purified protein was dialyzed against 20 mM Tris-HCl, 100 mM NaCl (pH 7.4) solution until use.

### Crystallization and structure determination

For crystallization, the purified *Lb2*Cas12a (10 mg/ml) and crRNA were mixed in a molar ratio of 1:2 and incubated at 4°C for 20 min. The *Lb2*Cas12a-crRNA complex and screening solution were mixed in 1:1 ratio and crystallized at 20°C by hanging drop vapor diffusion method. *Lb2*Cas12a-crRNA crystals were grown in 10 mM MgCl_2_, 0.1 M sodium cacodylate (pH 6.8), 17% PEG1000, and 1 mM DTT. The complex crystals were briefly soaked in cryoprotectant solution containing 25% w/v D- (+)-Glucose monohydrate and flash cooled at 100 K. Diffraction data were collected at the NSRRC, Taiwan TPS05A beamline at 0.99 Å wavelength. HKL2000 program [40] was used for data processing. The Matthews coefficient was 3.2 Å^3^/Da [41] with 61.55% solvent content and 2 molecules in the asymmetric unit. The complex structure of *Lb2*Cas12a-crRNA was determined by molecular replacement using Phenix-Phaser program [42] and the individual REC and NUC coordinates of *Lachnospiraceae bacterium ND2006 Lb*Cas12a (PDB: 5ID6) were used as the search models. Several rounds of model building were done using COOT program [43] followed by refinement using Phenix-Refine [44]. The final model had good stereochemistry, with 99.5% residues falling within the allowed regions of the Ramachandran plot. Although the average B factor is around 90Å^2^, the model has good electron density map well covering the model (S15 Fig). Molecular graphic images were prepared using CueMol2 and pymol program.

### Cryo-EM sample preparation and data collection

Purified *Lb2*Cas12a, crRNA with 20 nt guide sequences, and DNA were mixed in a ratio of 1:1.5:2 and remove excess nucleic acid by gel filtration chromatography. The fresh sample was purified at 0.5 mg/ml in buffer containing 20 mM HEPES-Na (pH 7), 150 mM NaCl, 10 mM MgCl_2_, 5 mM DTT. The cryo-EM data were collected at CBIS CryoEM Facility, National University of Singapore. Four microliters of sample were applied on glow-discharged UltrAufoil R1.2/1.3 (Quantifoil) and blotted for 1 s in 22°C with 100% humidity, a wait time of 15 s, a drain time of 0 s, and a force of −5 using FEI Vitrobot Marc IV. The grid was plunge-frozen in liquid ethane cooled by liquid nitrogen. The frozen-hydrated grid was loaded into Titan Krios cryo-electron microscope equipped with Gatan K3 direct-electron counting camera and operated at 300 keV, and 35-frame movies were collected at 81,000× magnification in counting mode with a physical pixel size of 1.105 Å/pixel. The images were recorded at defocus range of 0.5 to 2.5 μm. The exposure time was 3.49 s. The dose was 45 e/Å per movie stack. The 2,560 stacks of 35-frame movies were collected, using SerialEM program (FEI; Thermo Fisher Scientific).

### Image processing

The micrographs were pre-processed by Relion-3.1.1, and 2D- and 3D-classifications were done in cryoSPARC-3.2.0 [45]. The movie frames were aligned by MotionCor2, using Relion’s own implementation. Contrast transfer function was estimated by CTFFIND-4.1. The particles were first LoG-picked and then template-picked on the same set of micrographs. The duplicate picks were removed from the combined particle sets. The particles were extracted with box size 64^2^ pix^2^ by 4-fold binning (4.420 Å/pix) in Relion-3.1.1 and imported to cryoSPARC-3.2.0. Suboptimal particle images were removed by multiple rounds of 2D classification and class selection. The selected particles were exported to Relion-3.1.1 using pyem command csparc2star.py–copy-micrograph-coordinates and re-extracted with box size 128^2^ pix^2^ by 2-fold binning (2.210 Å/pix). The particle duplicates were removed by inter-particle 30 Å cutoff. The particles were again imported to cryoSPARC-3.2.0 and subjected to initial 3D modeling by Ab-initio Reconstruction with the number of models 3 and class similarity 0, followed by Heterogeneous Refinement. The particle set belonging to the least represented class was discarded. The particle sets belonging to remaining two 3D-classes were retained and subjected to another round of Heterogeneous Refinement using the previous three 3D-classes as the volume input. After 3 times Heterogeneous Refinement, the best 3D-class reaches 5.0 Å resolution (S11 and S12 Figs). The particles were exported again using csparc2star.py to Relion-3.1.1 and re-extracted with un-binned box size 256^2^ pix^2^ (1.105 Å/pix). The volume output belonging to the best 3D-class of Heterogeneous Refinement was rescaled to angpix 1.105 and re-sized to box size 256^3^ pix^3^ using relion_image_handler and imported to Relion-3.1.1 as 3D-reference. The re-extracted particles and the imported volume were subjected to 3D auto-refinement without masking. Following mask creation and post-processing, the images were Ctf-refined 3 times in order: (1) beam tilt; (2) anisotropic magnification; and (3) defocus per particle and astigmatism per micrograph. The images were Bayesian polished with the trained optimal parameters on the original output of MotionCorr2 in Relion-3.1.1. The polished particles were used for the second 3D-auto refinement without masking. The volume output from the first 3D-auto refinement was used as reference map with initial low-pass filter 40 Å. The volume from the second 3D auto-refinement was used to create a mask with low-pass filter 20 Å, initial binarization threshold 0.004 and extension of binary map threshold by 7 pixels, adding a soft edge of 8 pixels. The new mask and the output from the second 3D auto-refinement were subjected to post-processing with automatic sharpening, resulting in 4.28 Å map. The post-processing output was used for the second round of Ctf-refinement and Bayesian polishing as described above. The polished particles, the mask, and output volume from the second 3D auto-refinement before sharpening were subjected to the third 3D auto-refinement, which resulted in map resolution 4.49 Å before sharpening and 4.09 Å after sharpening. The second polished particles were imported to cryoSPARC-3.2.0 and performed Ab initio Reconstruction and Non-uniform Refinement without further particle sorting. The map quality was improved to 3.95 Å.

### *Lb2*Cas12a/crRNA/DNA model building

The initial model was prepared by using SWISS-MODEL online suite based on the structure 5XUS as a template. Phenix dock-in-map [46] was used for initial model building followed by manual model building in COOT [47] using the cryo-EM map. After initial model building, the model was refined against the EM-derived maps using the phenix.real-space-refinement tool from the PHENIX software package [46], employing rigid body, local grid, NCS, and gradient minimization. The model was used to sharpen the map in CCPEM-1.6.0, and further rebuilt by Flex-EM and ISOLDE-1.0.1. This model was then subjected to additional rounds of manual model-building and refinement which resulted in a final model-to-map cross-correlation coefficient of 0.72 for *Lb2*Cas12a/crRNA/DNA model. Stereo-chemical properties of the model were evaluated by Molprobity [48]. Molecular graphic images were prepared using CueMol2 (http://www.cuemol.org/en/) and Chimera programs [49].

### RNA substrate in vitro transcription

The primers were designed to amplify DNA fragments with T7 promotor. In vitro transcription was based on standard procedures of RiboMAX Large Scale RNA Production System T7 (Promega). The reaction mixture was further purified by 7% polyacrylamide 8 M urea denaturation TBE gel and classical ethanol precipitation [50].

### Dynamic light scattering (DLS)

The purified *Lb2*Cas12a or mutant was incubated with RNA, DNA, or RNA/DNA-duplex. Subsequently, the *apo* or complex was dialyzed against the pH 6.2, pH 7.4, and pH 8.0 buffers (20 mM Tris-HCl, 100 mM NaCl). Further purification was performed by size exclusion chromatography using a HiLoad 16/600 Superdex 200 prep grade preparative column (GE Healthcare). Subsequently, 5 μl sample (1 mg/ml) was used for DLS (DynaPro NanoStar) experiments that was run at 4°C.

### Electrophoretic mobility shift assay

*Lb2*Cas12a wild type or mutant (0–400 nM) was incubated with crRNA (100 nM) in the buffer that contained 20 mM HEPES 10 mM MgCl_2_, 100 mM NaCl, 1 mM DTT, 4% glycerol, pH 7.4 at 4°C for 15 min. Subsequently, the incubation mixture with native PAGE loading dye was used to perform 7% native polyacrylamide gel electrophoresis. The gel was stained with SYBR Safe at room temperature for 5 min and imaged via Imaging via ChemiDoc Imaging System (Bio-Rad Laboratories).

### dsDNA cleavage assay

To generate Biotin-tagged substrates, DNA duplex was amplified by PCR reaction using forward or reverse primers labeled with 5′ Biosg (synthesized from IDT). The 20 μl cleavage reaction mixture containing 20 mM Tris-HCl, 50 mM NaCl, 1 mM DTT, 10 mM MgCl_2_ (pH 7.4), 100 nM crRNA, 50 nM dsDNA, and 100 nM *Lb2*Cas12a WT or mutant was incubated at 37°C for 20 min. The reaction mixture was subjected to denaturation TBE-urea 7% PAGE and imaged using SYBR Safe stain and ChemiDoc MP Imaging System. The cleaved products were excised from the gel to perform sequencing analysis. The biotin-tagged cleavage products were detected by Chemiluminescent Nucleic Acid Detection Module (Thermo, 89880) and visualized by ChemiDoc Imaging System. The reaction was performed in independent triplicates. Oligonucleotides used in this assay are shown in S8 Table.

### ssDNA cleavage assay

Single-stranded DNA activity was detected using M13mp18 ssDNA (New England Biolabs) as the substrate. The RNA-DNA duplex-triggered ssDNA cleavage reaction was performed according to the previous protocols [17]. The Mn^2+^-triggered ssDNA cleavage reaction was performed as follows: the 20 μl reaction mixture containing 30 nM M13mp18 ssDNA, 20 nM *Lb2*Cas12a, 20 mM Tris-HCl, 50 mM NaCl, 1 mM DTT (pH 7.4), 10 mM MgCl_2_ or CaCl_2_ or MnCl_2_ or CuCl_2_ was incubated at 37°C for 15 min. The reaction mixture was separated by 1.2% agarose gel. The gel was stained with SYBR Gold and the image was captured with ChemiDoc Imaging System.

### 2D Classification of negative staining *apo Lb2*Cas12a particles and *Lb2*Cas12a-crRNA

The concentration of *apo Lb2*Cas12a and *Lb2*Cas12a-crRNA were diluted to 0.02 mg/ml to perform negative staining, 5 μl sample was loaded onto grid (carbon film 300 mesh copper, EMS) for 60 s and excess sample was removed with filter paper. Then, 5 μl stain solution (Uranyless EM Stain, EMS) was applied on the grid for 30 s and removed with filter paper. The grid was dried at room temperature for 10 min. The images were captured by Tecnai 12 (FEI 120kV, LaB6, magnification 52Kx).

### Trypsinization

Approximately 5 μg of purified *Lb2*Cas12a was incubated with crRNA at a molar ratio of 1:1 for 15 min on ice. Subsequently, 5 μg *apo Lb2*Cas12a and *Lb2*Cas12a-crRNA complex were incubated with 0.1 μg trypsin at room temperature in the 10 μl reaction buffer containing 30 mM HEPES, 150 mM NaCl, 1 mM DTT (pH 7.4), 10 mM MgCl_2_, 0.02 μg trypsin, 5 μg *apo Lb2*Cas12a, or *Lb2*Cas12a-crRNA complex. Five replicates were set up for each *apo Lb2*Cas12a and *Lb2*Cas12a-crRNA complex reaction mixture. The reaction was terminated at 1 min, 3 min, 5 min, 10 min, and 15 min by adding SDS-PAGE loading buffer and heating at 100°C for 2 min. The reaction product was resolved by SDS-PAGE gel and imaged with ChemiDoc Imaging System.

## Supporting information

S1 Fig crRNA-dependent double-stranded DNA cleavage by *Lb2*Cas12a. (A) dsDNA substrate cleavage by *Lb2*Cas12a with crRNA, TS: Target dsDNA; C: Control, dsDNA only; S: Substrate dsDNA; P: Cleaved products. (B) Cleavage site of target DNA analyzed by Sanger sequencing, black line: Canonical base pairs; gray line: noncanonical base pairs; red line: duplex base pairs. (C) dsDNA substrate cleavage by *Lb2Cas12a* with different metal ion, C: Control, dsDNA only; S: Substrate dsDNA, P: Cleaved products. (D) 5′-TTNN-3′ PAM nucleotide preferences of *Lb2*Cas12a. None: only target dsDNA, S: Substrate dsDNA, P: Cleaved products.
(TIF)

S2 Fig Recognition of the crRNA by *Lb*2Cas12a. (A) Representation of the *Lb2*Cas12a-bound crRNA. Cartoon shown in the electron density map (2mFo-DFc, gray for crRNA and blue for Mg^2+^ and H_2_O), contoured at 1.0 σ, base-pairs shown as stick representation. (B) Representation of the *Lb2*Cas12a-bound crRNA by cartoon, base-pairs shown in simple cartoon representation, Mg^2+^ and [H_2_O] are indicated by gray and green, respectively. (C) Schematic diagram of the interactions between the crRNA and *Lb2*Cas12a residues, residues that interact with the crRNA via their main chain are shown in parentheses.
(TIF)

S3 Fig Negative staining analysis of *apo Lb2*Cas12a and *Lb2*Cas12a-crRNA. (A) Left, representative raw negative staining micrograph and 2D class averages of *apo Lb2*Cas12a particles. Right, representative raw negative staining micrograph and 2D class averages of *Lb2*Cas12a-crRNA particles (B) *apo Lb2*Cas12a and *Lb2*Cas12a-crRNA complex were treated with trypsin and were resolved by SDS-PAGE.
(TIF)

S4 Fig Electrostatic surface potential of the *Lb2*Cas12a, *apo*, crRNA bound, crRNA DNA bound complex. (A) Electrostatic surface potential of the *apo Lb2*Cas12a (Crystal structure). (B) Electrostatic surface potential of the *Lb2*Cas12a-crRNA complex (Crystal structure). Zoomed view of RNA-binding pocket (inset) (C) Electrostatic surface potential of the *Lb2*Cas12a-crRNA-DNA complexes (Cryo-EM structure). Electrostatic surface transparency 0.3. Different domains are distinguished by label color.
(TIF)

S5 Fig crRNA-binding pocket in *Lb2*Cas12a-crRNA complex. (A) Interactions between REC lobe and NUC lobe in *Lb2*Cas12a-crRNA complex. Left: Crystal structure of *Lb2*Cas12a-crRNA in tube representation. Right: The close-up view of interaction between WED, RuvC domain, and REC lobe. The key residues are shown in sticks representation. (B) Left, the crRNA binding pocket is located between WED and RuvC domains. The ribbon transparency 0.4. Right, crRNA-binding pocket displayed with surface. The key residues are shown in sticks representation. The surface transparency 0.4.
(TIF)

S6 Fig Gel filtration chromatography analysis of the wild-type and mutants *Lb2*Cas12a. (A) Gel filtration chromatography analysis of the *apo Lb2*Cas12a under different pH environments. (B) Gel filtration chromatography analysis of the *apo*, RNA-bound, and RNA/DNA-bound *Lb2*Cas12a. (C) Gel filtration chromatography analysis of R864E/K866E/R868E/K869E mutant in *apo* and RNA-bound forms.
(TIF)

S7 Fig Dynamic light scattering analysis of the wild type and mutants of *Lb2*Cas12a. (A–C) Dynamic light scattering analysis of the *apo Lb2*Cas12a under different pH environments. (D, E) Dynamic light scattering analysis of RNA-bound and RNA/DNA-bound *Lb2*Cas12a. (F, G) Dynamic light scattering analysis of R864E/K866E/R868E/K869E mutant in *apo* and RNA-bound forms. The data underlying S7 Fig can be found in S1 Data.
(TIF)

S8 Fig EMSA of wild type and mutants of *Lb2*Cas12a. WT: Wild Type, ΔF558 ~ T660: the truncation of *Lb2*Cas12a that removed PI domain. R864E/K866E/R868E/K869E: the variant that breaks the interaction between REC lobe and NUC lobe. L696A, Q886A, and Q887A: the variants that regulate crRNA into the RNA-binding pocket.
(TIF)

S9 Fig REC lobe in *Lb2*Cas12a. (A) Comparison of the REC lobes between the *Lb2*Cas12a-crRNA and *Lb2*Cas12a-crRNA-DNA. Different domains are distinguished by color. The edge of REC1 of *Lb2*Cas12a-crRNA-DNA is indicated crimson. The edge of REC2 of *Lb2*Cas12a-crRNA-DNA is indicated chartreuse. (B) Superposition of NUC lobe of *apo* (Crystal structure), RNA-bound (Crystal structure), and RNA/DNA-bound (Cryo-EM structure) *Lb*2Cas12a. Different domains are distinguished by color. NUC lobe transparency 0.5. The edge of REC lobes of *Lb2*Cas12a in *apo*, RNA-bound and RNA/DNA-bound forms are indicated with gold, fuchsia, and blue, respectively.
(TIF)

S10 Fig Identification of PAM by PI domain. (A) Comparison of the PI domain between the *Lb2*Cas12a-crRNA and *Lb2*Cas12a-crRNA-DNA. (B) Left: Representation of the PI domain and PAM of cryo-EM *Lb2*Cas12a/crRNA/DNA. Cartoon model fit in the density map (ccp4, gray for density), contoured at 1.0 σ. PAM base-pairs and Lys571 are shown in stick representation. Right: Residues Lys575 and Lys518 are involved in the recognition of TTTN PAM. (C) dsDNA cleavage activity analysis of mutants in PAM recognition region. S: target dsDNA substrate, P: cleavage product, N: negative control, dsDNA only.
(TIF)

S11 Fig Overview of cryo-EM data processing workflow for 3D reconstruction. The value beneath the map is the number of particles put in to build the map. In Heterogeneous Refinement, consistent B = −100 is applied.
(TIF)

S12 Fig Local resolution distribution and map quality estimates of the final NU-Refinement. **(A)** Local resolution distribution. **(B)** Angular distribution at the last iteration. **(C)** FSC curve from NU-Refinement by FSC = 0.143 cutoff.
(TIF)

S13 Fig Conserved catalytic residues for dsDNA and ssDNA cleavage. (A) Conserved catalytic residues for DNA cleavage in *apo*Cas12a-crRNA (left) and *Lb2*Cas12a-crRNA (right). (B) Catalytic residues for dsDNA cleavage, C: Control, dsDNA only, S: Substrate dsDNA, P: Cleaved products. (C) Left, catalytic residues for ssDNA cleavage triggered by crRNA-DNA duplex; middle, catalytic residues for ssDNA cleavage triggered Mn^2+^; right, Mn^2+^-mediated ssDNA cleavage, C: Control, M13mp18 ssDNA only, S: Substrate ssDNA, P: Cleaved products.
(TIF)

S14 Fig Sequence alignment of *Lb2*Cas12a with other Cas12a orthologs from different organisms. Multiple sequence alignment by Clustal Omega (https://www.ebi.ac.uk/Tools/msa/clustalo/) and ESPript 3.0 (http://espript.ibcp.fr/ESPript/cgi-bin/ESPript.cgi). Cas12a proteins sequences from species *Lachnospiraceae bacterium MA2020*, *Lachnospiraceae bacterium ND2006*, *Moraxella bovoculi*, *Acidaminococcus* sp. *BV3L6*, *Francisella tularensis subsp*. *novicida U112*, respectively. The secondary structure elements are shown above the sequence base on the structure of *Lb2*Cas12a-crRNA. The domains are shown below the sequence. Arrows indicate catalytic amino acid residues involved function.
(ZIP)

S15 Fig 2Fo-Fc.map of Crystal structure of *apo Lb2*Cas12a Ser304-Phe307. Representation of the *apo Lb2*Cas12a Ser304-Phe307. Sticks shown in the electron density map (ccp4, gray for density), contoured at 1.0 σ. The average B-factor parameters of Ser304, Ala305, Phe 306, and Phe 307 atoms were 91 **Å**^**2**^, 114 **Å**^**2**^, 102 **Å**^**2**^, and 103 **Å**^**2**^, respectively.
(TIF)

S1 Table Crystallographic data collection and refinement statistics. (PDF)

S2 Table Structures of Cas12a orthologs. (PDF)

S3 Table Structural homologs of REC lobe of *apo Lb2*Cas12a from the DALI server. (PDF)

S4 Table Structural homologs of NUC lobe of *apo Lb2*Cas12a from the DALI server (Top 20). (PDF)

S5 Table Structural homologs of *Lb2*Cas12a-crRNA from the DALI server (Top 20). (PDF)

S6 Table Independent domains superpositions in *apo Lb2*Cas12a and *Lb2*Cas12acrRNA. (PDF)

S7 Table cryo-EM data collection and refinement statistics. (PDF)

S8 Table Oligonucleotides used for preparation of target GFP sequences for PAM Identification. (PDF)

S1 Video Cartoon model showing dynamic *apo Lb2*Cas12a and crRNA binding. The conformational changes induced by crRNA binding are shown in this order: (1) Overall dynamic of *apo Lb2*Cas12a; (2) the process of crRNA binding; (3) stabilization of *Lb2*Cas12a-crRNA in compact conformation; (4) Met493~Leu523 undergoing major conformational changes. The different domains are distinguished by color. The backbone of RNA is highlighted in black. The video was made by UCSF Chimera.
(MP4)

S1 Data Raw data of dynamic light scattering analysis of *Lb2*Cas12a. Dynamic light scattering analysis of the *Lb2*Cas12a or mutants under different pH environments; different RNA-bound state.
(XLSX)

S1 Raw Images **Image 1. Recognition of the crRNA by *Lb2*Cas12a**. dsDNA cleavage activity analysis of *Lb2*Cas12a mutants in RNA recognition region. C: Control; only target dsDNA, S: Substrate, P: Cleaved product. This figure relates to the Fig 2C. **Image 2. The guide strand length of crRNA required for *Lb2*Cas12a to achieve cleavage activity**. None: only target dsDNA, S: target dsDNA substrate, P: cleavage product. This figure relates to the Fig 4C. **Image 3. crRNA-dependent double-stranded DNA cleavage by *Lb2*Cas12a**. dsDNA substrate cleavage by *Lb2*Cas12a with crRNA, TS: Target dsDNA; C: Control, dsDNA only; S: Substrate dsDNA; P: Cleaved products. This figure relates to the S1A Fig. **Image 4. dsDNA substrate cleavage by *Lb2Cas12a* with different metal ion**. C: Control, dsDNA only; S: Substrate dsDNA, P: Cleaved products. This figure relates to the S1C Fig. **Image 5. 5′-TTNN-3′ PAM nucleotide preferences of *Lb2*Cas12a**. None: only target dsDNA, S: Substrate dsDNA, P: Cleaved products. This figure relates to the S1D Fig. **Image 6. Trypsinization of *apo Lb2*Cas12a and *Lb2*Cas12a-crRNA complex**. *apo Lb2*Cas12a and *Lb2*Cas12a-crRNA complex were treated with trypsin and were resolved by SDS-PAGE. This figure relates to the S3B Fig. **Image 7. EMSA of wild type and mutants of *Lb2*Cas12a**. WT: Wild type, ΔF558 ~ T660: the truncation of *Lb2*Cas12a that removed PI domain. R864E/K866E/R868E/K869E: the variant that breaks the interaction between REC lobe and NUC lobe. L696A, Q886A, and Q887A: the variants that regulate crRNA into the RNA-binding pocket. This figure relates to the S8 Fig. **Image 8. dsDNA cleavage activity analysis of mutants in PAM recognition region**. S: target dsDNA substrate, P: cleavage product, N: negative control, dsDNA only. This figure relates to the S10C Fig. **Image 9. Catalytic residues for dsDNA cleavage**. C: Control, dsDNA only, S: Substrate dsDNA, P: Cleaved products. This figure relates to the S13B Fig. **Image 10. ssDNA cleavage of *Lb2*Cas12a**. Left, catalytic residues for ssDNA cleavage triggered by crRNA-DNA duplex; middle, catalytic residues for ssDNA cleavage triggered Mn^2+^; right, Mn^2+^-mediated ssDNA cleavage, C: Control, M13mp18 ssDNA only, S: Substrate ssDNA, P: Cleaved products. This figure relates to the S13C Fig.
(PDF)

## Abbreviations

BH: Bridge Helix
DETECTR: DNA Endonuclease Targeted CRISPR Trans Reporter
DLS: dynamic light scattering
dsDNA: double-stranded DNA
FRET: fluorescence resonance energy transfer
NHEJ: nonhomologous end-joining
PAM: protospacer adjacent motif
PISA: Protein Interfaces, Surfaces and Assemblies
