# Supplementary figures and images for: Structures of apo Cas12a and its complex with crRNA and DNA reveal the dynamics of ternary complex formation and target DNA cleavage
Source: PLoS Biol. 2023 Mar 14;21(3):e3002023. doi: 10.1371/journal.pbio.3002023 (PMC10013913; doi:10.1371/journal.pbio.3002023)

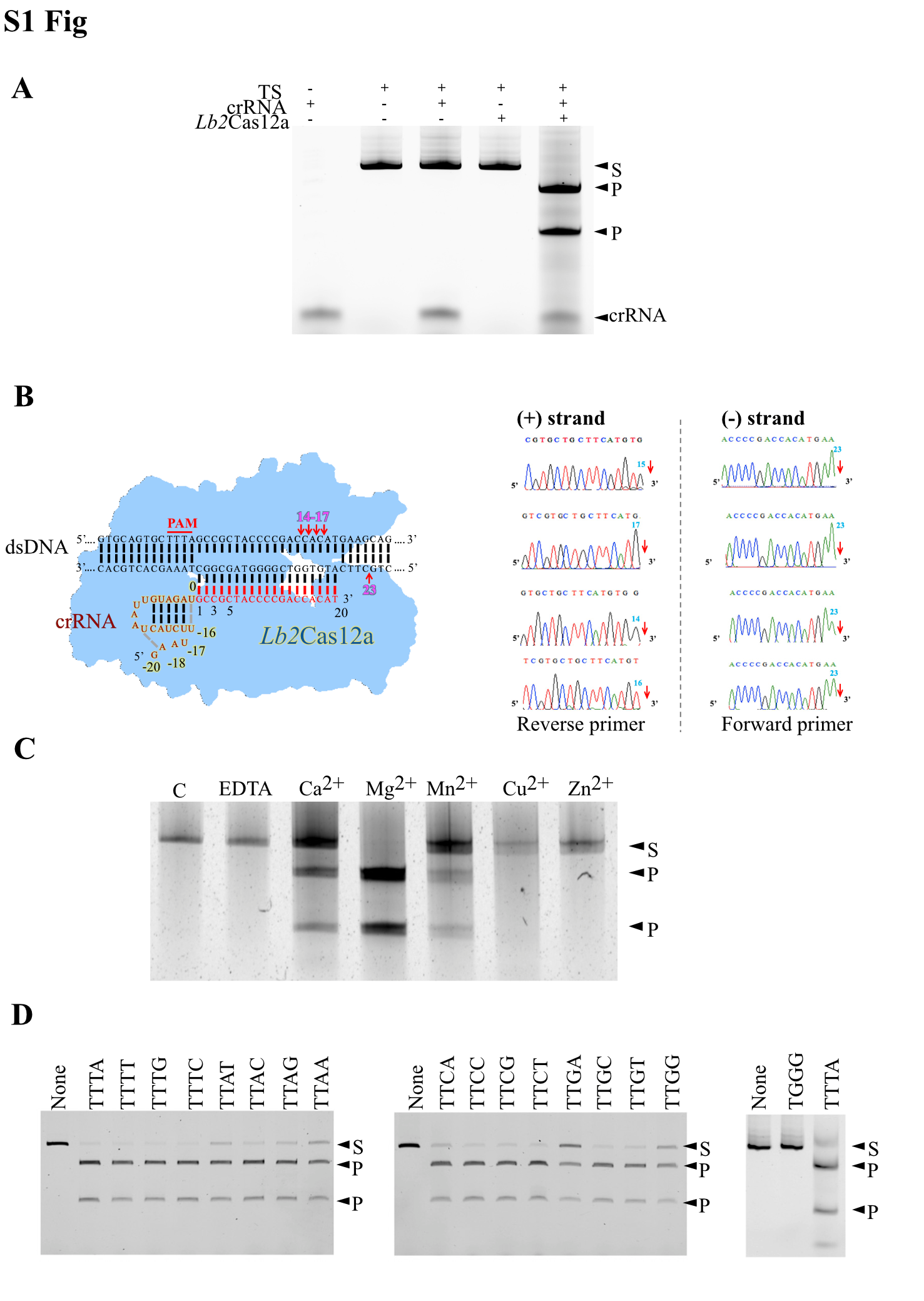

Supplement: S1 Fig — (A) dsDNA substrate cleavage by Lb2Cas12a with crRNA, TS: Target dsDNA; C: Control, dsDNA only; S: Substrate dsDNA; P: Cleaved products. (B) Cleavage site of target DNA analyzed by Sanger sequencing, black line: Canonical base pairs; gray line: noncanonical base pairs; red line: duplex base pairs. (C) dsDNA substrate cleavage by Lb2Cas12a with different metal ion, C: Control, dsDNA only; S: Substrate dsDNA, P: Cleaved products. (D) 5′-TTNN-3′ PAM nucleotide preferences of Lb2Cas12a. None: only target dsDNA, S: Substrate dsDNA, P: Cleaved products. (TIF) [file pbio.3002023.s001.tif]

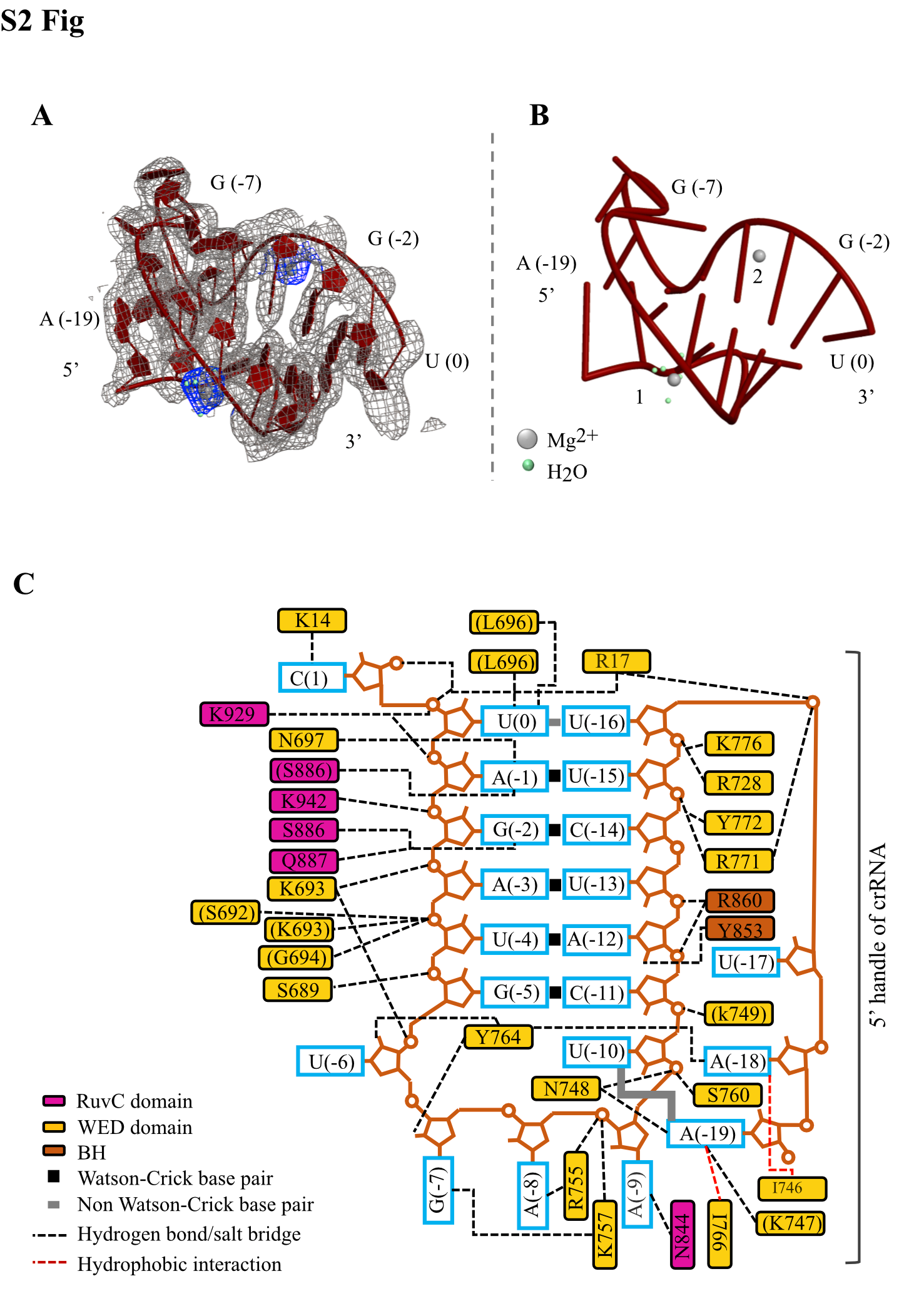

Supplement: S2 Fig — (A) Representation of the Lb2Cas12a-bound crRNA. Cartoon shown in the electron density map (2mFo-DFc, gray for crRNA and blue for Mg2+ and H2O), contoured at 1.0 σ, base-pairs shown as stick representation. (B) Representation of the Lb2Cas12a-bound crRNA by cartoon, base-pairs shown in simple cartoon representation, Mg2+ and [H2O] are indicated by gray and green, respectively. (C) Schematic diagram of the interactions between the crRNA and Lb2Cas12a residues, residues that interact with the crRNA via their main chain are shown in parentheses. (TIF) [file pbio.3002023.s002.tif]

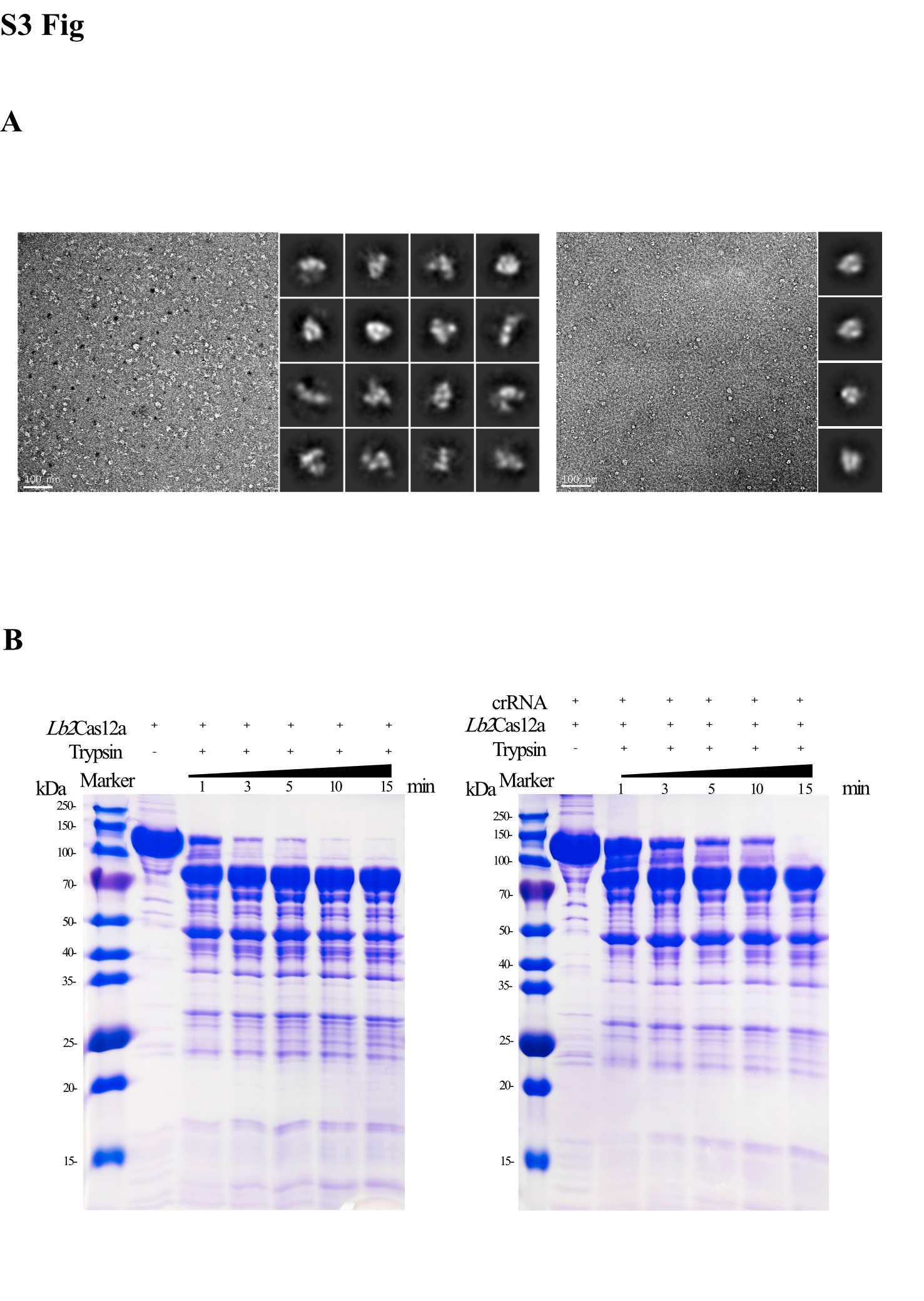

Supplement: S3 Fig — (A) Left, representative raw negative staining micrograph and 2D class averages of apo Lb2Cas12a particles. Right, representative raw negative staining micrograph and 2D class averages of Lb2Cas12a-crRNA particles (B) apo Lb2Cas12a and Lb2Cas12a-crRNA complex were treated with trypsin and were resolved by SDS-PAGE. (TIF) [file pbio.3002023.s003.tif]

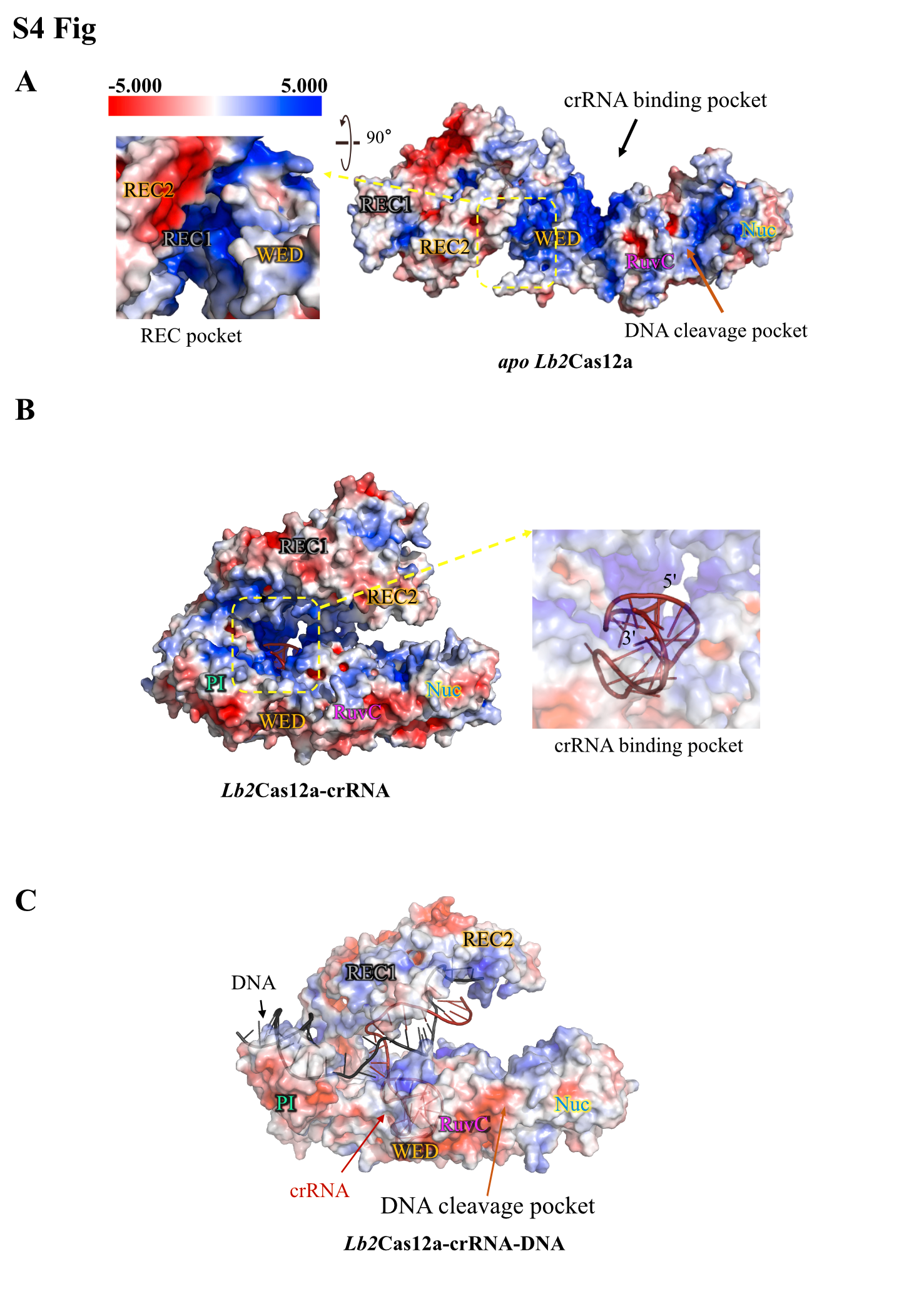

Supplement: S4 Fig — (A) Electrostatic surface potential of the apo Lb2Cas12a (Crystal structure). (B) Electrostatic surface potential of the Lb2Cas12a-crRNA complex (Crystal structure). Zoomed view of RNA-binding pocket (inset) (C) Electrostatic surface potential of the Lb2Cas12a-crRNA-DNA complexes (Cryo-EM structure). Electrostatic surface transparency 0.3. Different domains are distinguished by label color. (TIF) [file pbio.3002023.s004.tif]

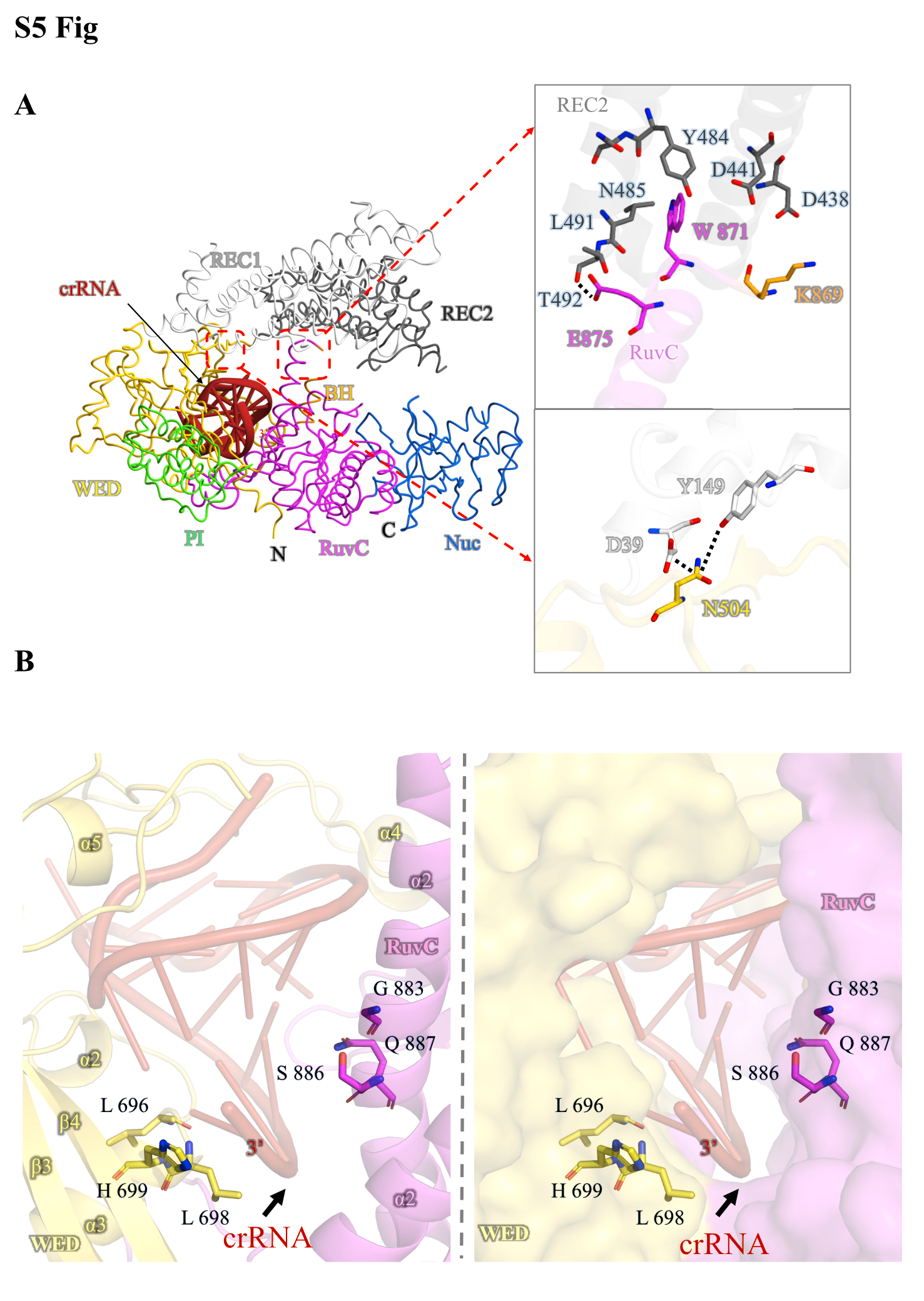

Supplement: S5 Fig — (A) Interactions between REC lobe and NUC lobe in Lb2Cas12a-crRNA complex. Left: Crystal structure of Lb2Cas12a-crRNA in tube representation. Right: The close-up view of interaction between WED, RuvC domain, and REC lobe. The key residues are shown in sticks representation. (B) Left, the crRNA binding pocket is located between WED and RuvC domains. The ribbon transparency 0.4. Right, crRNA-binding pocket displayed with surface. The key residues are shown in sticks representation. The surface transparency 0.4. (TIF) [file pbio.3002023.s005.tif]

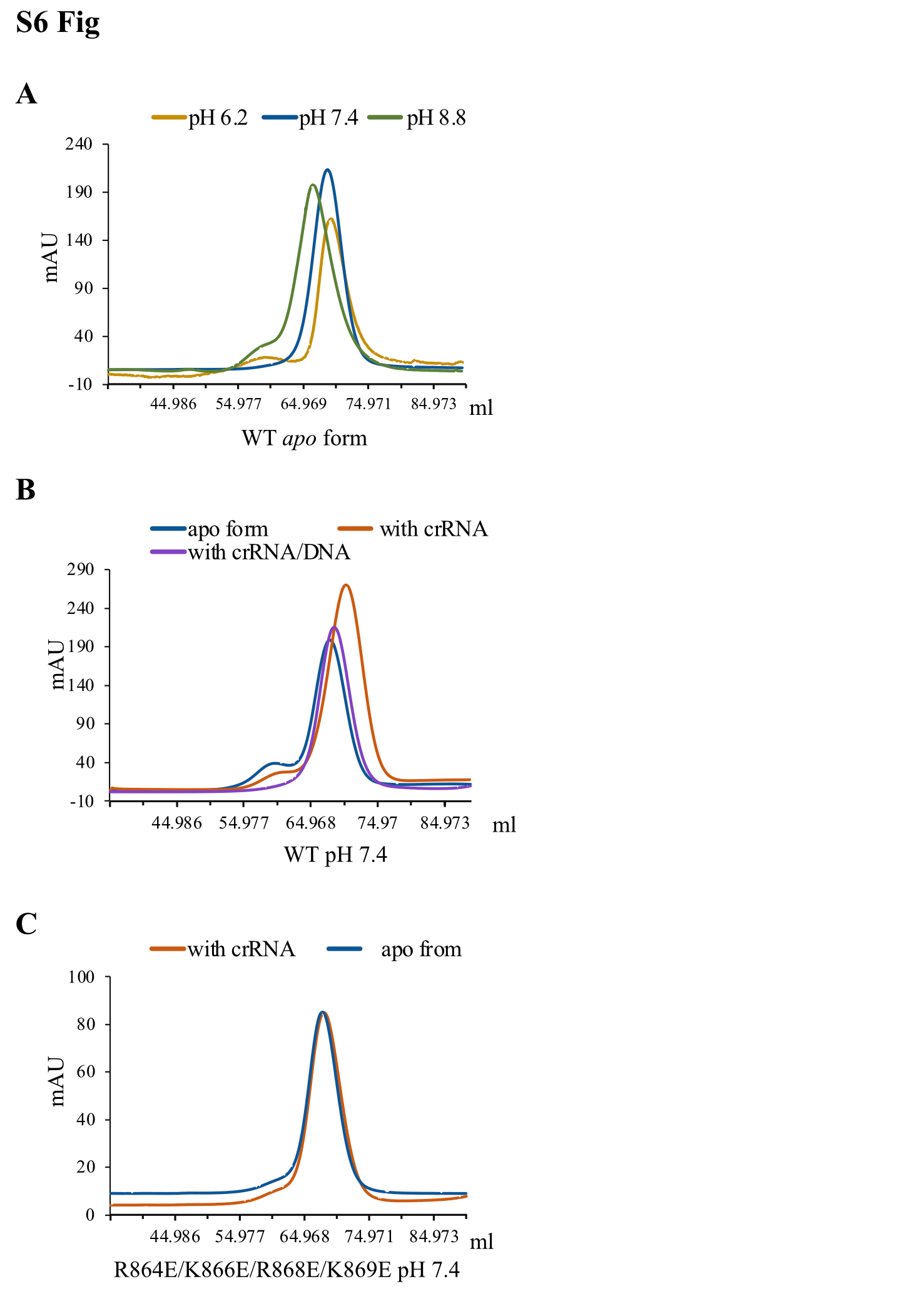

Supplement: S6 Fig — (A) Gel filtration chromatography analysis of the apo Lb2Cas12a under different pH environments. (B) Gel filtration chromatography analysis of the apo, RNA-bound, and RNA/DNA-bound Lb2Cas12a. (C) Gel filtration chromatography analysis of R864E/K866E/R868E/K869E mutant in apo and RNA-bound forms. (TIF) [file pbio.3002023.s006.tif]

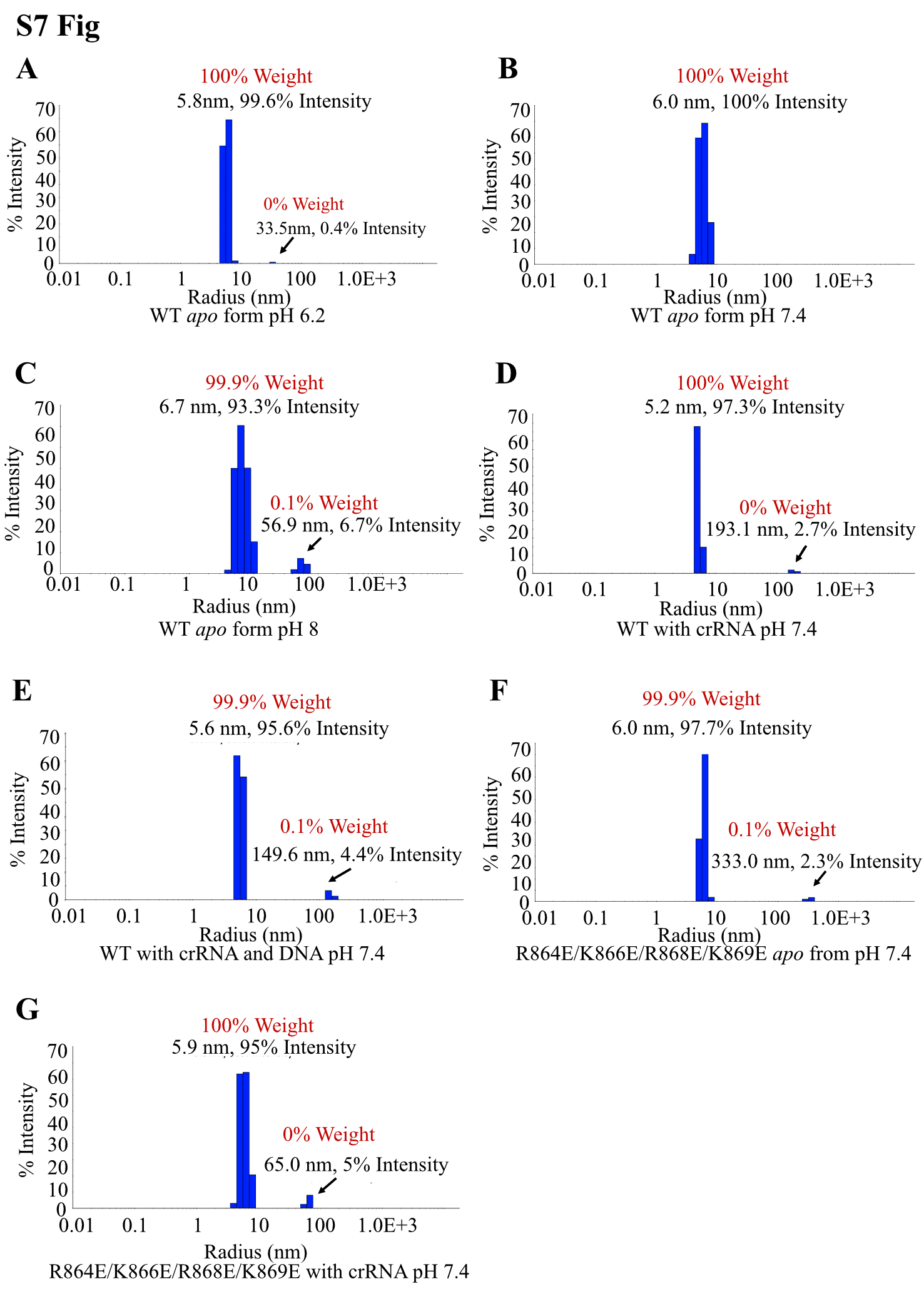

Supplement: S7 Fig — (A–C) Dynamic light scattering analysis of the apo Lb2Cas12a under different pH environments. (D, E) Dynamic light scattering analysis of RNA-bound and RNA/DNA-bound Lb2Cas12a. (F, G) Dynamic light scattering analysis of R864E/K866E/R868E/K869E mutant in apo and RNA-bound forms. The data underlying S7 Fig can be found in S1 Data. (TIF) [file pbio.3002023.s007.tif]

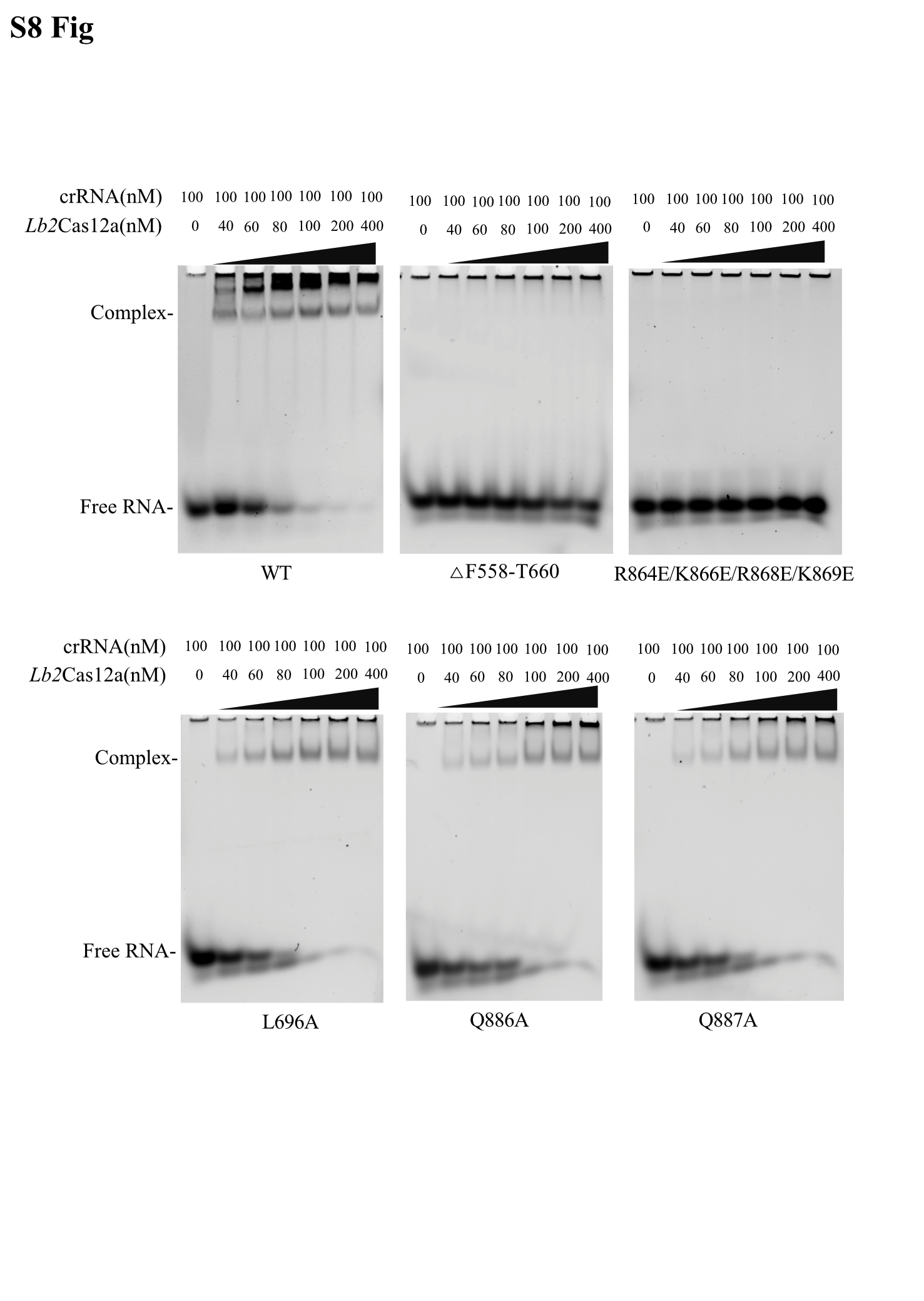

Supplement: S8 Fig — WT: Wild Type, ΔF558 ~ T660: the truncation of Lb2Cas12a that removed PI domain. R864E/K866E/R868E/K869E: the variant that breaks the interaction between REC lobe and NUC lobe. L696A, Q886A, and Q887A: the variants that regulate crRNA into the RNA-binding pocket. (TIF) [file pbio.3002023.s008.tif]

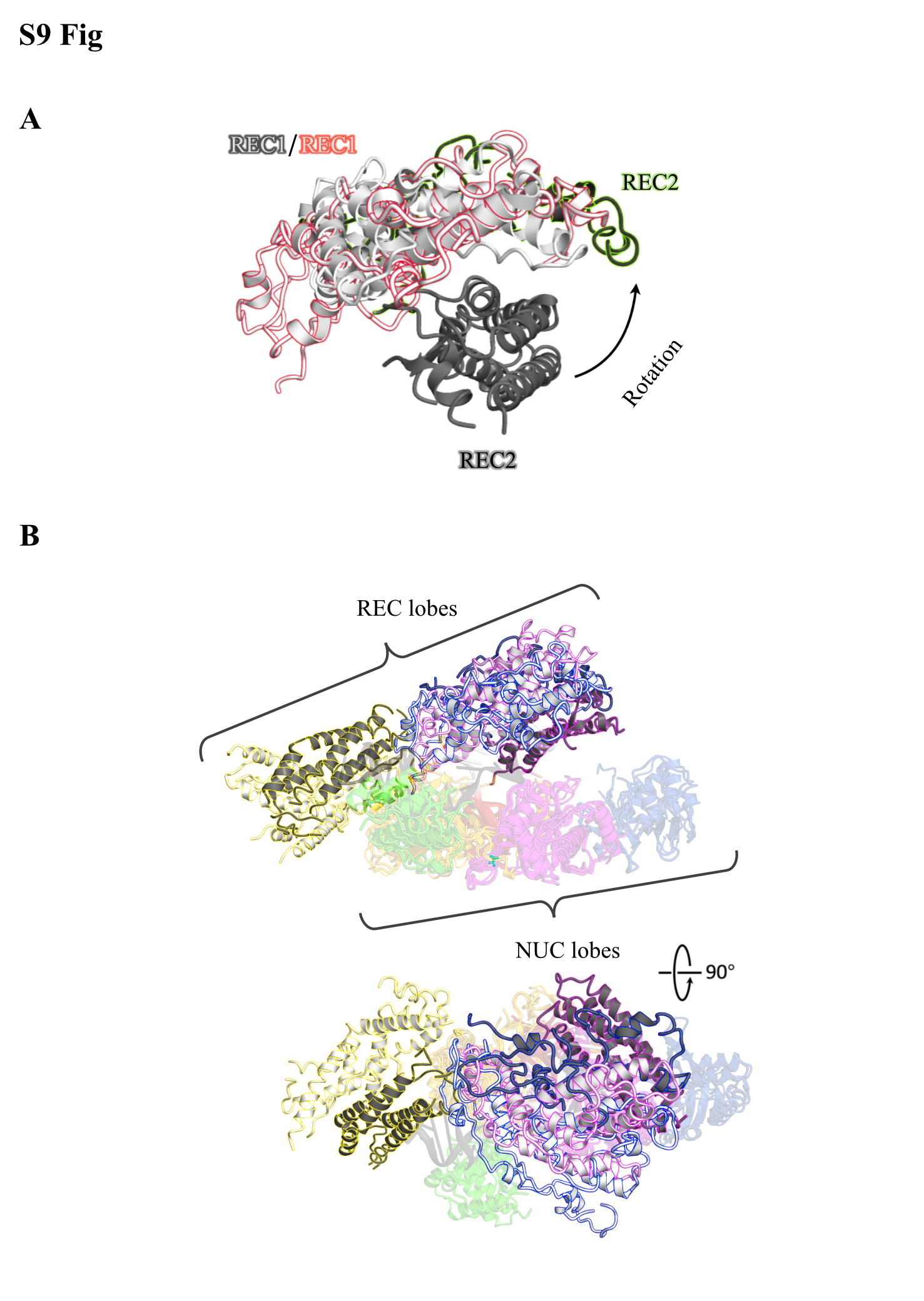

Supplement: S9 Fig — (A) Comparison of the REC lobes between the Lb2Cas12a-crRNA and Lb2Cas12a-crRNA-DNA. Different domains are distinguished by color. The edge of REC1 of Lb2Cas12a-crRNA-DNA is indicated crimson. The edge of REC2 of Lb2Cas12a-crRNA-DNA is indicated chartreuse. (B) Superposition of NUC lobe of apo (Crystal structure), RNA-bound (Crystal structure), and RNA/DNA-bound (Cryo-EM structure) Lb2Cas12a. Different domains are distinguished by color. NUC lobe transparency 0.5. The edge of REC lobes of Lb2Cas12a in apo, RNA-bound and RNA/DNA-bound forms are indicated with gold, fuchsia, and blue, respectively. (TIF) [file pbio.3002023.s009.tif]

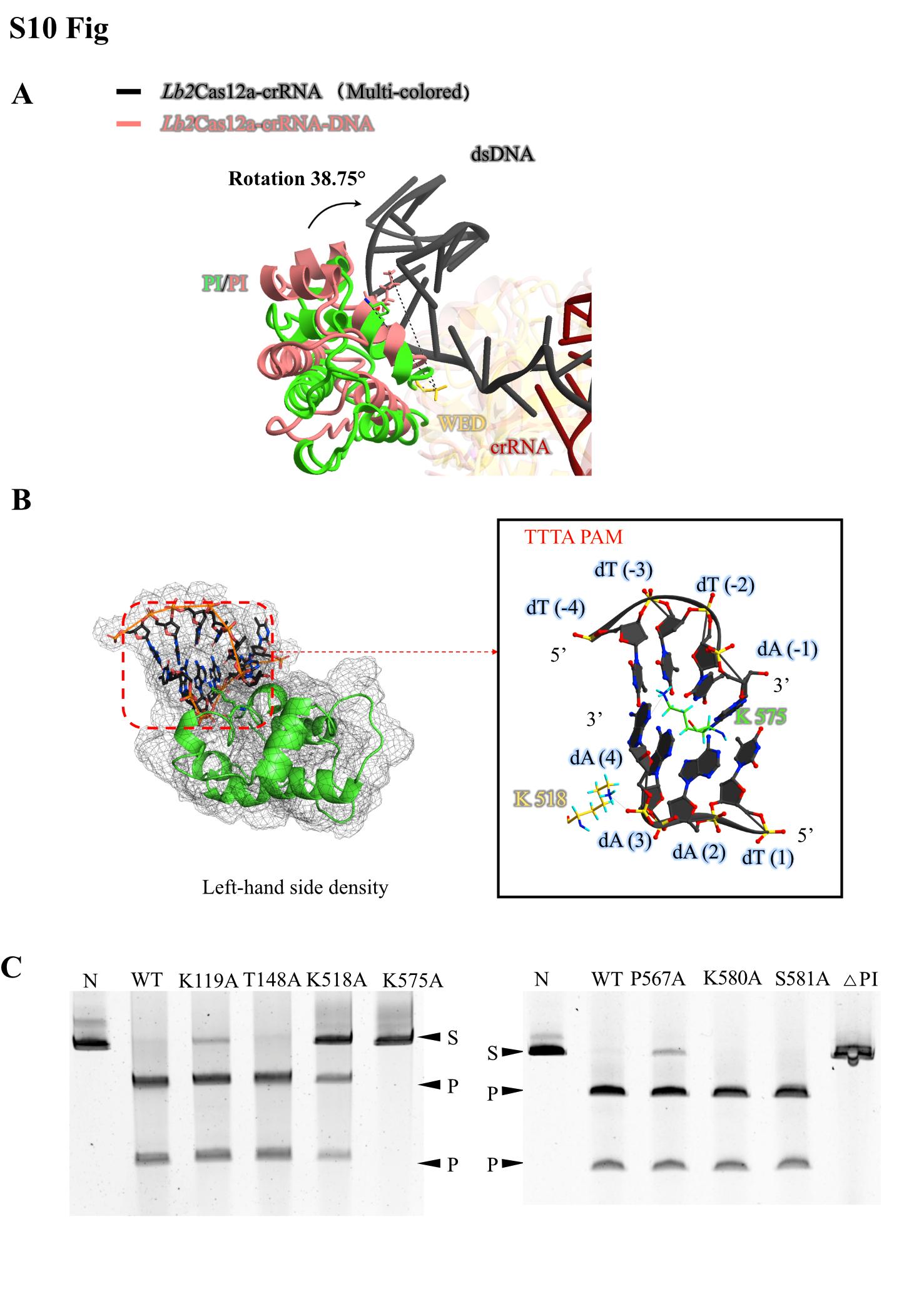

Supplement: S10 Fig — (A) Comparison of the PI domain between the Lb2Cas12a-crRNA and Lb2Cas12a-crRNA-DNA. (B) Left: Representation of the PI domain and PAM of cryo-EM Lb2Cas12a/crRNA/DNA. Cartoon model fit in the density map (ccp4, gray for density), contoured at 1.0 σ. PAM base-pairs and Lys571 are shown in stick representation. Right: Residues Lys575 and Lys518 are involved in the recognition of TTTN PAM. (C) dsDNA cleavage activity analysis of mutants in PAM recognition region. S: target dsDNA substrate, P: cleavage product, N: negative control, dsDNA only. (TIF) [file pbio.3002023.s010.tif]

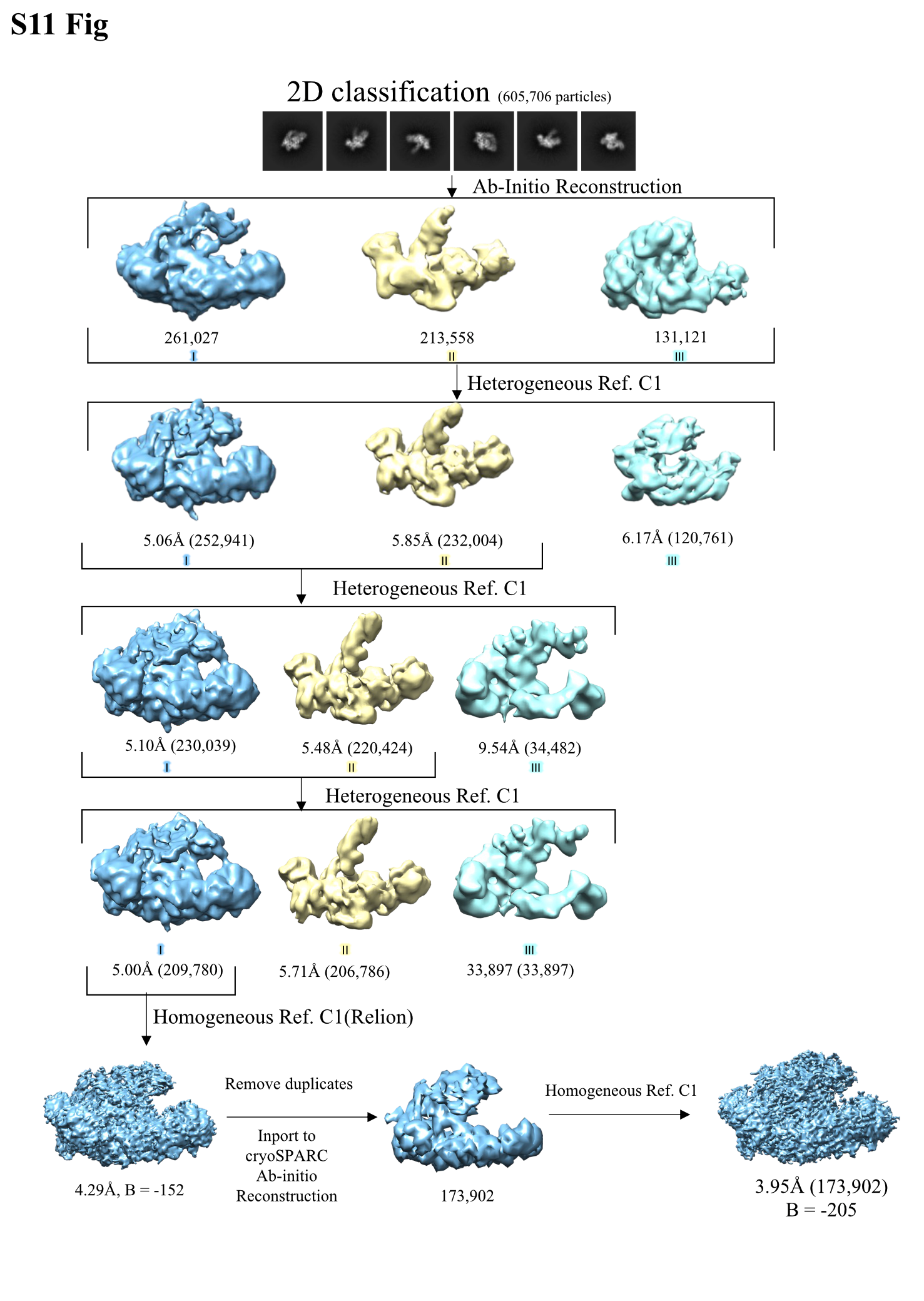

Supplement: S11 Fig — The value beneath the map is the number of particles put in to build the map. In Heterogeneous Refinement, consistent B = −100 is applied. (TIF) [file pbio.3002023.s011.tif]

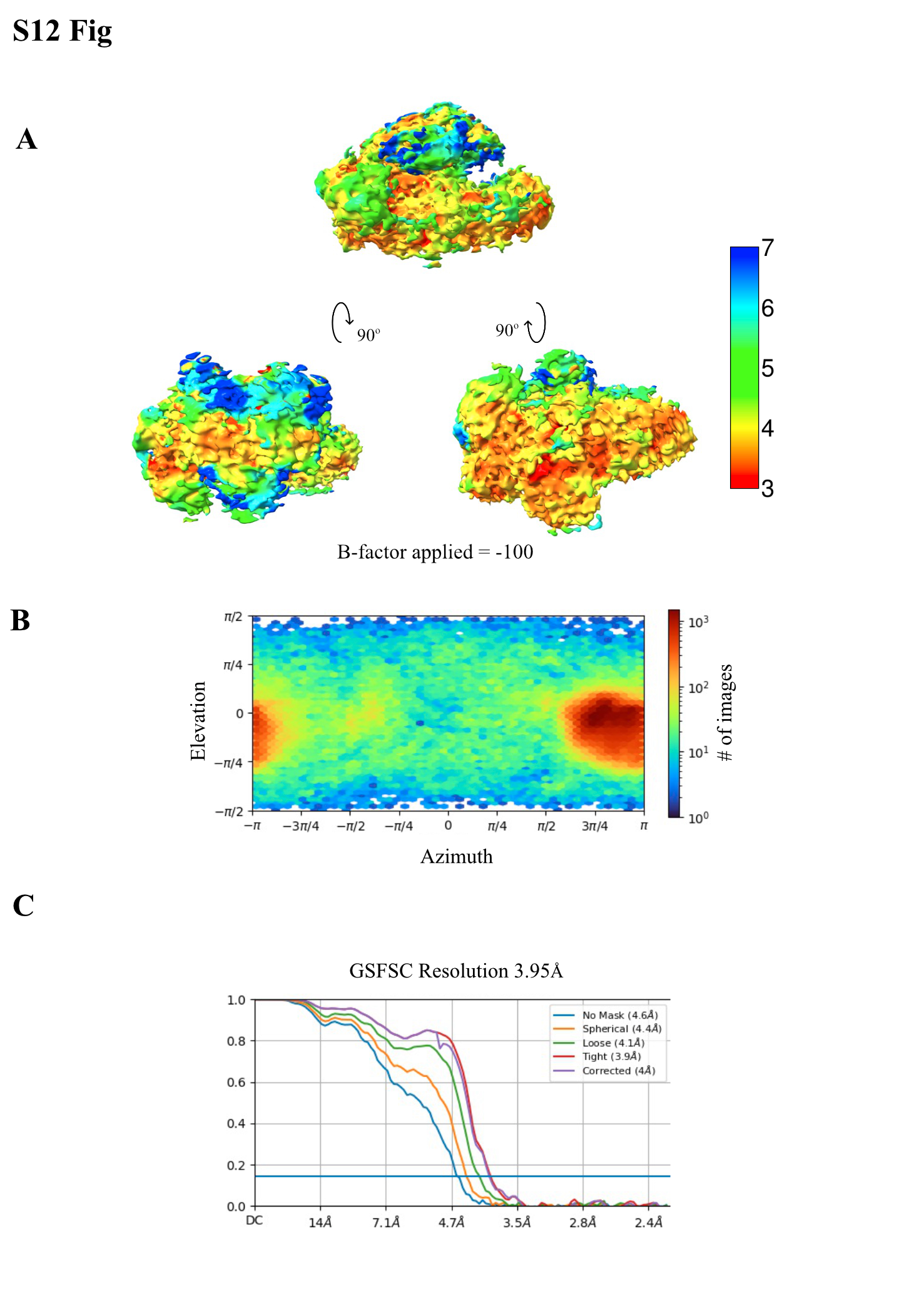

Supplement: S12 Fig — (A) Local resolution distribution. (B) Angular distribution at the last iteration. (C) FSC curve from NU-Refinement by FSC = 0.143 cutoff. (TIF) [file pbio.3002023.s012.tif]

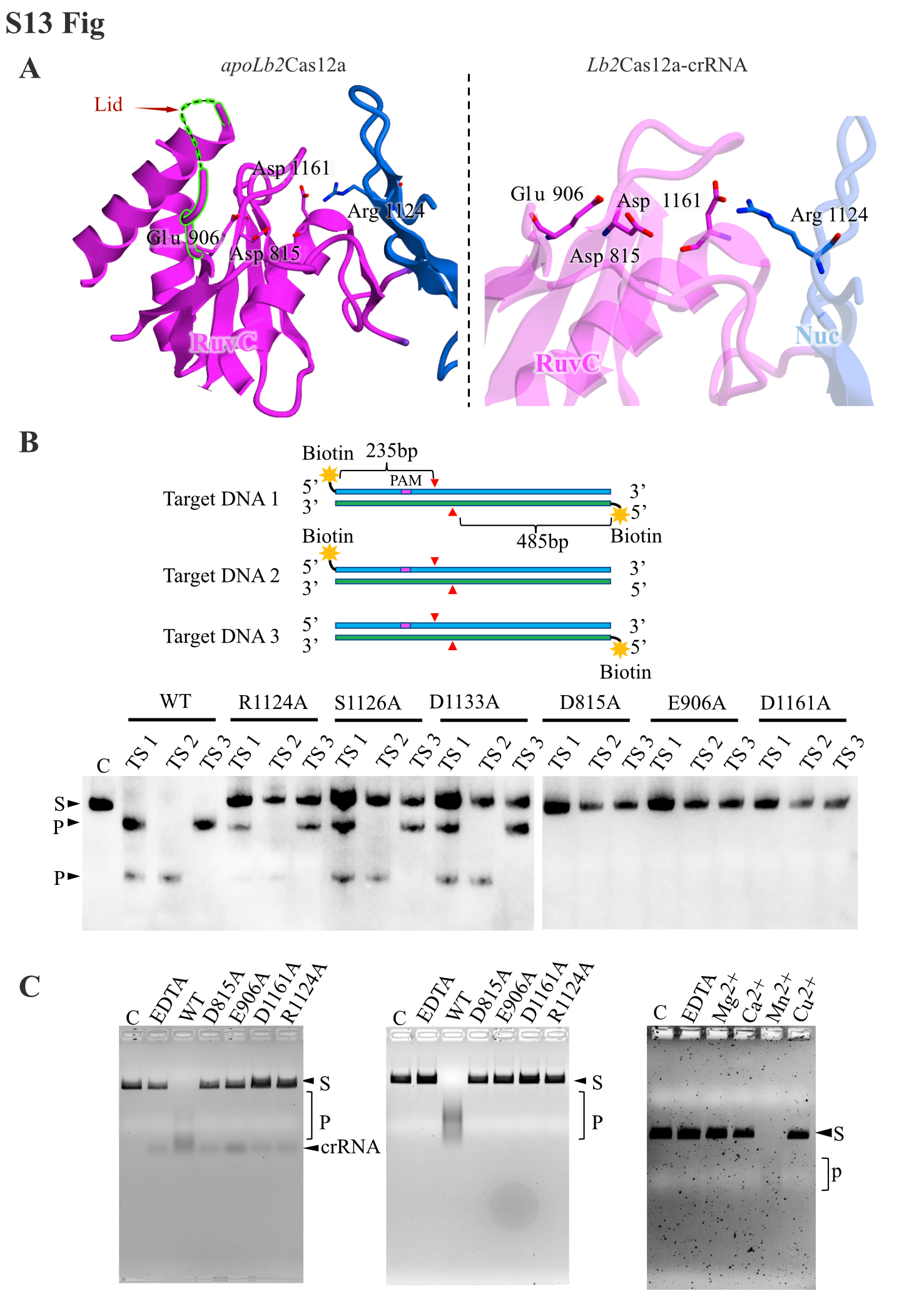

Supplement: S13 Fig — (A) Conserved catalytic residues for DNA cleavage in apoCas12a-crRNA (left) and Lb2Cas12a-crRNA (right). (B) Catalytic residues for dsDNA cleavage, C: Control, dsDNA only, S: Substrate dsDNA, P: Cleaved products. (C) Left, catalytic residues for ssDNA cleavage triggered by crRNA-DNA duplex; middle, catalytic residues for ssDNA cleavage triggered Mn2+; right, Mn2+-mediated ssDNA cleavage, C: Control, M13mp18 ssDNA only, S: Substrate ssDNA, P: Cleaved products. (TIF) [file pbio.3002023.s013.tif]

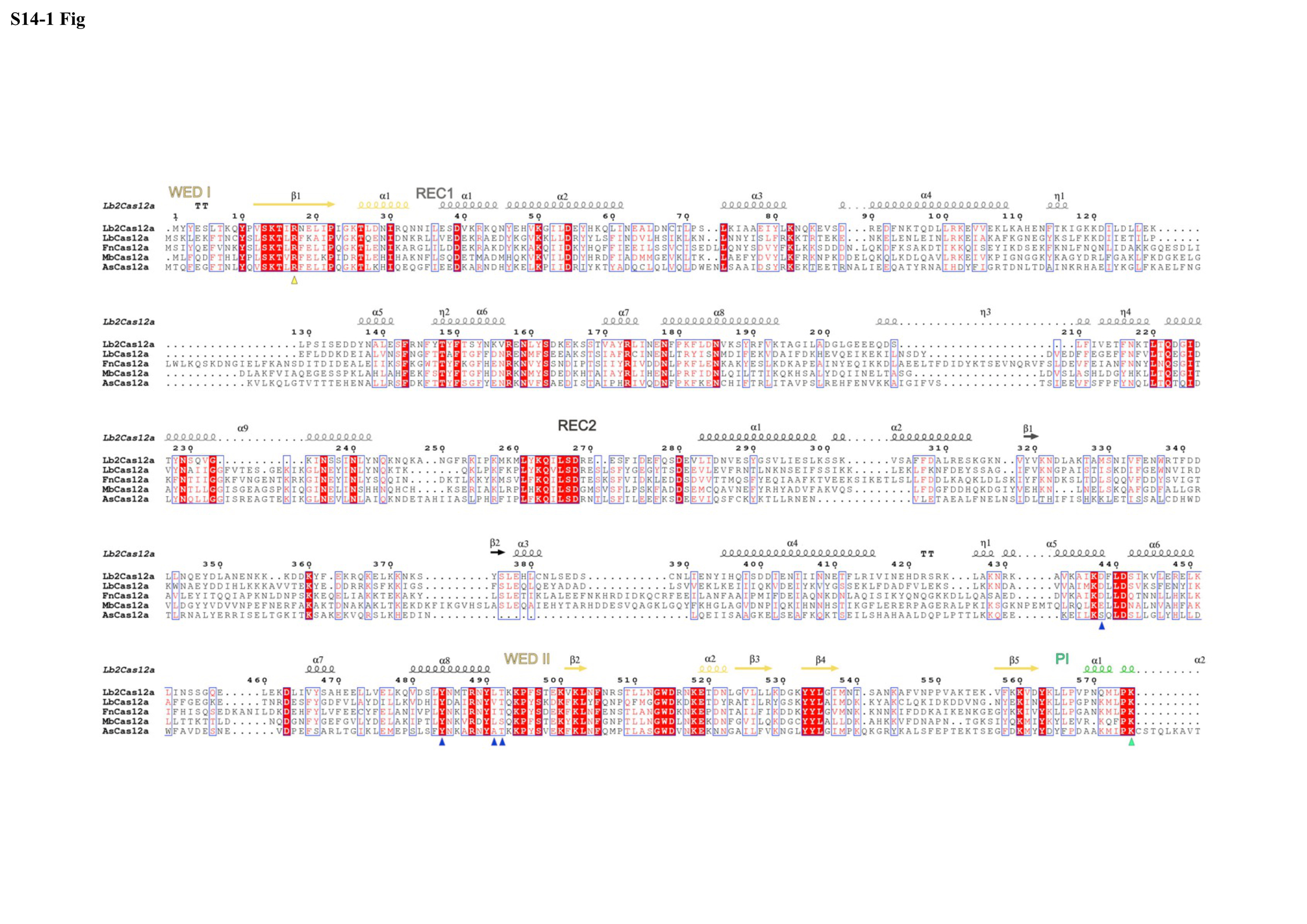

Supplement: S14 Fig — Multiple sequence alignment by Clustal Omega (https://www.ebi.ac.uk/Tools/msa/clustalo/) and ESPript 3.0 (http://espript.ibcp.fr/ESPript/cgi-bin/ESPript.cgi). Cas12a proteins sequences from species Lachnospiraceae bacterium MA2020, Lachnospiraceae bacterium ND2006, Moraxella bovoculi, Acidaminococcus sp. BV3L6, Francisella tularensis subsp. novicida U112, respectively. The secondary structure elements are shown above the sequence base on the structure of Lb2Cas12a-crRNA. The domains are shown below the sequence. Arrows indicate catalytic amino acid residues involved function. (ZIP) [file pbio.3002023.s014.zip › S14-1_Fig.tif]

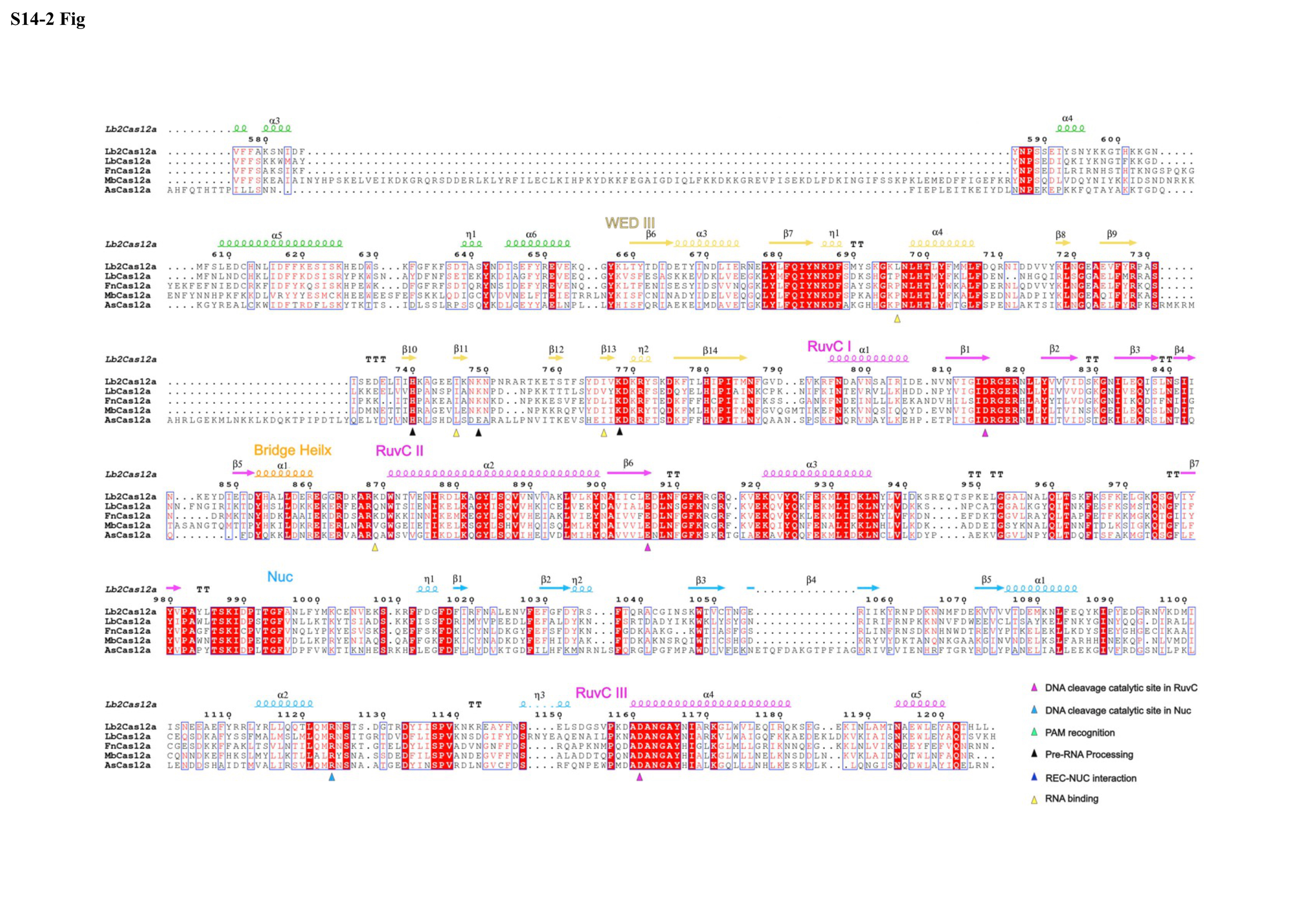

Supplement: S14 Fig — Multiple sequence alignment by Clustal Omega (https://www.ebi.ac.uk/Tools/msa/clustalo/) and ESPript 3.0 (http://espript.ibcp.fr/ESPript/cgi-bin/ESPript.cgi). Cas12a proteins sequences from species Lachnospiraceae bacterium MA2020, Lachnospiraceae bacterium ND2006, Moraxella bovoculi, Acidaminococcus sp. BV3L6, Francisella tularensis subsp. novicida U112, respectively. The secondary structure elements are shown above the sequence base on the structure of Lb2Cas12a-crRNA. The domains are shown below the sequence. Arrows indicate catalytic amino acid residues involved function. (ZIP) [file pbio.3002023.s014.zip › S14-2_Fig.tif]

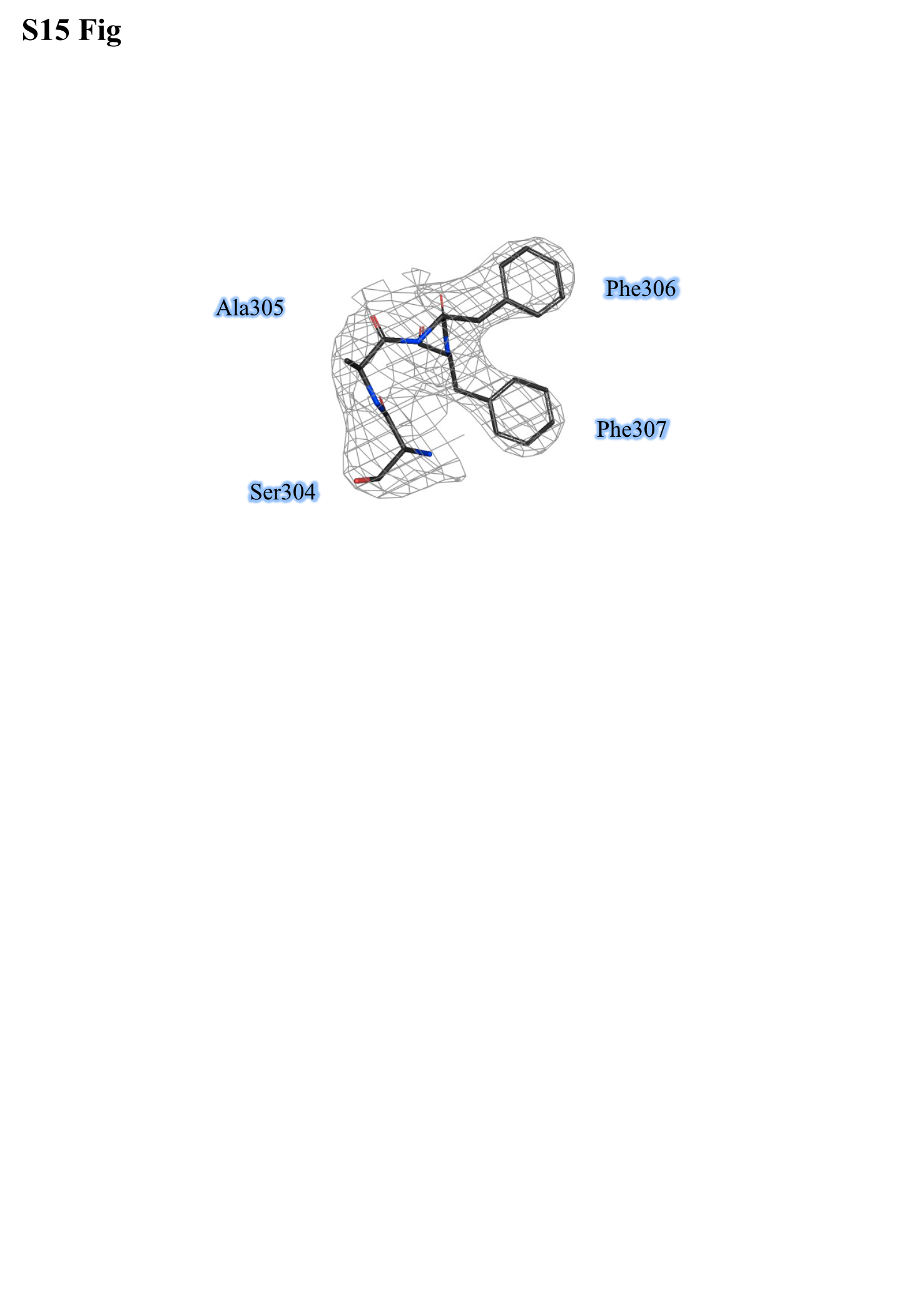

Supplement: S15 Fig — Representation of the apo Lb2Cas12a Ser304-Phe307. Sticks shown in the electron density map (ccp4, gray for density), contoured at 1.0 σ. The average B-factor parameters of Ser304, Ala305, Phe 306, and Phe 307 atoms were 91 Å2, 114 Å2, 102 Å2, and 103 Å2, respectively. (TIF) [file pbio.3002023.s015.tif]
