# Supplementary material for: Structures of apo Cas12a and its complex with crRNA and DNA reveal the dynamics of ternary complex formation and target DNA cleavage
Source: PLoS Biol. 2023 Mar 14;21(3):e3002023. doi: 10.1371/journal.pbio.3002023 (PMC10013913; doi:10.1371/journal.pbio.3002023)
Supplement: S1 Table — (PDF) [file pbio.3002023.s016.pdf]

Table. S1 Data collection and refinement statistics

		*Lb2*Cas12a-crRNA
**Data collection**		
Beamline		NSRRC TPS05A
Space group		C121
Wavelength (Å)		0.99
Cell dimensions	a, b, c (Å)	235.70, 139.30, 110.15
	α, β, γ (°)	85.62, 90.00, 90.00
Molecules/ASU		2
Observed reflections		336648
Unique reflections		47506
Resolution (Å)*		50.0-3.0 (3.1-3.0))
Rsym (%)[a]		0.16 (0.93)
I/s(I)		15.60 (1.92)
Completeness (%)[a]		99.83 (99.40)
Redundancy[a]		6.60 (5.40)
**Refinement**		
Resolution range (Å)		29.98-3.10
R_{work} (R_{free}) (%)		23.52(25.25)
B-factors (Å^2)	Protein (residues 2052)	99.13
	RNA (nucleotide 21)	72.68
	Mg ion (2)	60.77
	Water (6)	64.36
R.m.s. deviations	Bond lengths (Å)	0.004
	Bond angles (°)	0.79
Ramachandran-plot statistics (%)	Favoured regions	95.34
	Allowed regions	4.14
	Disallowed regions	0.52
