# Supplementary material for: Structures of apo Cas12a and its complex with crRNA and DNA reveal the dynamics of ternary complex formation and target DNA cleavage
Source: PLoS Biol. 2023 Mar 14;21(3):e3002023. doi: 10.1371/journal.pbio.3002023 (PMC10013913; doi:10.1371/journal.pbio.3002023)
Supplement: S2 Table — (PDF) [file pbio.3002023.s017.pdf]

Table. S2 Structures of Cas12a orthologs

**Cas12a**	***Apo* Cas12a**	**Cas12a/crRNA**	**Cas12a/crRNA/DNA**
***Lb2*Cas12a Current study structures**	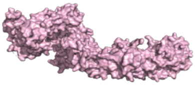	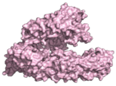	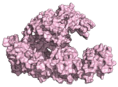
*Lb*Cas12a	Not available	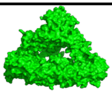 5ID6	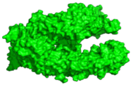 5XUS
*Fn*Cas12a	Not available	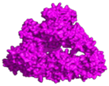 5NG6A	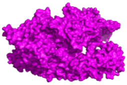 6GTG
*As*Cas12a	Not available	Not available	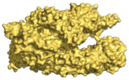 5B43
*Mb*Cas12a	Not available	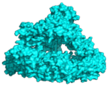 6IV6	Not available
