# Supplementary material for: Structures of apo Cas12a and its complex with crRNA and DNA reveal the dynamics of ternary complex formation and target DNA cleavage
Source: PLoS Biol. 2023 Mar 14;21(3):e3002023. doi: 10.1371/journal.pbio.3002023 (PMC10013913; doi:10.1371/journal.pbio.3002023)
Supplement: S3 Table — (PDF) [file pbio.3002023.s018.pdf]

Table S3. Structural homologs of REC lobe of *apo Lb2*Cas12a from the DALI server
(Top 20)

No	Chain	Z	Rmsd (Å)	lali	nres	%id	Description
1	6nme-A	22.9	3.4	333	1193	30	CPF1
2	6nmd-A	22.0	4.8	323	1202	29	CPF1
3	6nmc-A	21.5	4.7	321	1202	30	CPF1
4	5xuz-E	21.5	3.4	240	1208	31	LBCPF1
5	5xuu-A	21.5	4.4	252	1213	31	LBCPF1
6	5xus-A	21.4	4.3	255	1206	30	LBCPF1
7	6p7n-E	21.2	3.1	331	1070	28	CAS12A
8	6p7n-A	20.5	3.1	333	1070	28	CAS12A
9	5xuz-A	20.3	3.3	244	1216	30	LBCPF1
10	6nma-B	20.0	3.6	340	1206	29	ANTI-CRISPR VA4/CAS12A
11	6nm9-B	19.8	3.5	337	1205	29	ANTI-CRISPR VA4/CAS12A
12	6nm9-D	19.7	3.6	339	1205	29	ANTI-CRISPR VA4/CAS12A
13	6omv-B	19.3	4.4	249	1208	30	CAS12a
14	6kl9-A	19.2	3.7	337	1180	30	LBCAS12A
15	5nfv-A	19.1	3.2	232	1258	28	CPF1
16	6i1l-D	19.1	3.3	232	1254	28	CAS12A
17	6i1k-A	19.1	3.4	231	1282	29	CAS12A
18	6i1l-A	18.9	3.3	236	1277	29	CAS12A
19	6gtc-A	18.7	3.5	343	1280	28	CAS12A
20	5mga-A	18.6	3.9	250	1180	27	CPF1

Lali: The number of residues aligned
Nres: The number of residues in the target structure
