# Supplementary material for: Structures of apo Cas12a and its complex with crRNA and DNA reveal the dynamics of ternary complex formation and target DNA cleavage
Source: PLoS Biol. 2023 Mar 14;21(3):e3002023. doi: 10.1371/journal.pbio.3002023 (PMC10013913; doi:10.1371/journal.pbio.3002023)
Supplement: S4 Table — (PDF) [file pbio.3002023.s019.pdf]

Table S4. Structural homologs of NUC lobe of *apo Lb2*Cas12a from the DALI server

(Top 20)

No	Chain	Z	Rmsd (Å)	lali	nres	%id	Description
1	5xuu-A	43.8	2.1	551	1213	45	LBCPF1
2	5xh7-A	42.9	2.6	543	1283	41	CRISPR-CPF1
3	5xuz-E	42.9	2.1	547	1208	45	LBCPF1
4	6omv-B	42.3	2.2	551	1208	44	CPF1
5	6nmc-A	42.2	2.2	551	1202	45	CPF1
6	5id6-A	42.1	2.4	557	1209	43	CPF
7	6i1k-A	42	2.5	544	1282	49	CRISPR-CAS12A
8	6nm9-B	41.8	2.2	553	1205	45	ANTI-CRISPR VA1/CAS12A
9	6nma-B	39.5	2.2	553	1206	45	ANTI-CRISPR VA1/CAS12A
10	6p7n-A	39	1.9	511	1070	45	ANTI-CRISPR VA4/CAS12A
11	6nmd-A	38.3	2.2	551	1202	44	CPF1
12	6i1l-D	34.3	2	540	1254	48	CRISPR-CAS12A
13	5xus-A	33	2.1	548	1206	45	LBCPF1
14	5xuz-A	32.8	2	548	1216	45	LBCPF1
15	6nme-A	32.2	2.4	547	1193	44	CPF1
16	5xh6-A	30.7	2.6	543	1282	41	CRISPR-CPF1
17	5nfv-A	29.8	2.9	547	1258	48	CRISPR-CPF1
18	6gtg-A	29.2	2.5	555	1298	48	CRISPR-CAS12A
19	6iv6-A	28.8	2.3	557	1209	49	NUCLEASE
20	6p7m-A	26.7	1.9	511	1070	45	CAS12A

Lali: The number of residues aligned
Nres: The number of residues in the target structure
