# Supplementary material for: Structures of apo Cas12a and its complex with crRNA and DNA reveal the dynamics of ternary complex formation and target DNA cleavage
Source: PLoS Biol. 2023 Mar 14;21(3):e3002023. doi: 10.1371/journal.pbio.3002023 (PMC10013913; doi:10.1371/journal.pbio.3002023)
Supplement: S5 Table — (PDF) [file pbio.3002023.s020.pdf]

Table. S5 Structural homologs of *Lb2*Cas12a-crRNA from the DALI server
(Top 20)

No	Chain	Z	Rmsd (Å)	lali	nres	% id	Description
1	6omv-B	30.7	4.3	983	1208	43	CAS12A
2	6nmd-A	30.3	3.8	1016	1202	44	CPF1
3	6nmc-A	30.0	3.8	1013	1202	44	CPF1
4	6i1k-A	29.8	6.7	923	1282	44	CRISPR CAS12A
5	6p7n-A	29.7	2.9	959	1070	41	ANTI-CRISPR VA4/CAS12a
6	5xuz-E	29.4	4.6	889	1208	44	LBCPF1
7	5id6-A	29.4	3.2	1084	1209	42	CPF1
8	5xuu-A	29.0	4.4	871	1213	44	LBCPF1
9	5xh7-A	28.9	5.0	871	1283	36	CRISPR CPF1
10	5xut-A	28.7	5.2	917	1217	43	LBCPF1
11	6nma-B	28.1	3.1	1070	1206	43	ACRVA1/CAS12A
12	6p7m-A	27.7	2.9	960	1070	41	CAS12A
13	6nm9-B	27.3	3.1	1072	1205	43	ACRVA4
14	6p7n-E	27.3	2.9	959	1070	41	ANTI-CRISPR VA4/CAS12A
15	6nm9-D	27.3	3.1	1071	1205	43	ACRVA4
16	6i1l-D	26.7	4.5	891	1254	45	CRISPR CAS12A
17	6gtc-A	26.0	3.4	1086	1280	44	CRISPR CAS12A
18	6iv6-A	25.9	3.3	1054	1209	43	NUCLEASE
19	6nme-A	25.9	3.2	1064	1193	44	CPF1
20	5xuz-A	25.6	5.2	898	1216	43	CAS12A

Lali: The number of residues aligned
Nres: The number of residues in the target structure
