# Supplementary material for: Structures of apo Cas12a and its complex with crRNA and DNA reveal the dynamics of ternary complex formation and target DNA cleavage
Source: PLoS Biol. 2023 Mar 14;21(3):e3002023. doi: 10.1371/journal.pbio.3002023 (PMC10013913; doi:10.1371/journal.pbio.3002023)
Supplement: S6 Table — (PDF) [file pbio.3002023.s021.pdf]

Table. S6 Independent domains superpositions in *apo Lb2*Cas12a and *Lb2*Cas12a-crRNA

No	Domain	Rmsd (Å)	No. of Cα atoms
1	REC1	0.722	171
2	REC2	0.816	98
3	WED	0.956	123
4	RuvC	0.788	175
5	Nuc	0.845	121
6	REC lobe	1.752	251
7	NUC lobe	1.348	482
