# Supplementary material for: Structures of apo Cas12a and its complex with crRNA and DNA reveal the dynamics of ternary complex formation and target DNA cleavage
Source: PLoS Biol. 2023 Mar 14;21(3):e3002023. doi: 10.1371/journal.pbio.3002023 (PMC10013913; doi:10.1371/journal.pbio.3002023)
Supplement: S7 Table — (PDF) [file pbio.3002023.s022.pdf]

Table. S7 cryo-EM data collection and refinement statistics

	Lb2Cas12a-crRNA-DNA
**Data collection**	
EM equipment	Titan Krios
Voltage (kV)	300 keV
Detector	Gatan K3
Pixel size (Å)	1.105 Å
Exposure time (s)	3.50
Dose rate (e^-/px/s)	6.65
Defocus range (µm)	0.5 to 2.5
Number of micrographs	2,560
Number of frames/images	35-frame
**Reconstruction**	
Software	CryoSPARC and Relion
Number of particles used	173,902
Final resolution (Å)	3.95
Symmetry	C1
**Model building and composition**	
Software	Phenix, Coot
Protein residues	1104
Ligands (Nucleotide)	67
Refinement Software	Phenix
CC_mask	0.72
CC_box	0.85
**Validation (proteins)**	
MolProbity score	2.22
Clash score, all atoms	10.38
Good rotamers (%)	100
**Ramachandran-plot statistics (%)**	
Favored (overall)	88.1
Allowed (overall)	11.9
Outlier (overall)	0
**R.m.s. deviations**	
Bond length (Å)	0.004
Bond angle (˚)	0.674
