# Supplementary material for: Structures of apo Cas12a and its complex with crRNA and DNA reveal the dynamics of ternary complex formation and target DNA cleavage
Source: PLoS Biol. 2023 Mar 14;21(3):e3002023. doi: 10.1371/journal.pbio.3002023 (PMC10013913; doi:10.1371/journal.pbio.3002023)
Supplement: S8 Table — (PDF) [file pbio.3002023.s023.pdf]

Table. S8 Oligonucleotides used for preparation of target GFP sequences for PAM identification

Identification	Sequences (5′-3′)
	*Lb2*Cas12a amplification
*Lb2*Cas12a-HindIII-F	CCAAGCTTATGTACTATGAGTCCCTG
*Lb2*Cas12a-Xho I-R	CCCTCGAGTTACAGCAGGTGTGTCTG
	In vitro transcription of RNA substrates
*Lb2*Cas12a-*GFP*-g-F	TAATACGACTCACTATAGGGGAATTTCTACTATTGTAGATAGCCGCTACCCCGACCACAT
*Lb2*Cas12a-*GFP*-g-R	ATGTGGTCGGGGTAGCGGCTATCTACAATAGTAGAAATTCCCCTATAGTGAGTCGTATTA
*Lb2*Cas12a-*M13phage*-g-F	TAATACGACTCACTATAGGGGAATTTCTACTATTGTAGATGGGCTATCAGTTCGCGCATT
*Lb2*Cas12a-*M13phage*-g-R	AATGCGCGAACTGATAGCCCATCTACAATAGTAGAAATTCCCCTATAGTGAGTCGTATTA
	Target GFP dsDNA
GFP-F	ATGGTGAGCAAGGGCGAGGA
GFP-R	TTACTTGTACAGCTCGTCCA
GFP-Biotin-F	BiosgATGGTGAGCAAGGGCGAGGA
GFP-Biotin-R	BiosgTTACTTGTACAGCTCGTCCA
	Cryo-EM Target DNA
Target strand	GCTTCATGTGGTCGGGGTAGCGGCTAAAGCACTG
Non-Target strand	CAGTGCTTTAGCCGCTACCCCGACCACATGAAGC
	PAM identification
PAM-TTTA-F	TGCAGTGCTTTAGCCGCTACCCCGACCACA
PAM-TTTA-R	TAGCGGCTAAAGCACTGCACGCCGTAGGTC
PAM-TTTT-F	GTGCAGTGCTTTTGCCGCTACCCCGACCAC
PAM-TTTT-R	TAGCGGCAAAAGCACTGCACGCCGTAGGTC
PAM-TTTC-F	GTGCAGTGCTTTCGCCGCTACCCCGACCAC
PAM-TTTC-R	TAGCGGCGAAAGCACTGCACGCCGTAGGTC
PAM-TTTG-F	GTGCAGTGCTTTGGCCGCTACCCCGACCAC
PAM-TTTG-R	TAGCGGCCAAAGCACTGCACGCCGTAGGTC
PAM-TTAT-F	GTGCAGTGCTTATGCCGCTACCCCGACCAC
PAM-TTAT-R	TAGCGGCATAAGCACTGCACGCCGTAGGTC
PAM-TTAC-F	GTGCAGTGCTTACGCCGCTACCCCGACCAC
PAM-TTAC-R	TAGCGGCGTAAGCACTGCACGCCGTAGGTC
PAM-TTAG-F	GTGCAGTGCTTAGGCCGCTACCCCGACCAC
PAM-TTAG-R	TAGCGGCCTAAGCACTGCACGCCGTAGGTC
PAM-TTAA-F	GTGCAGTGCTTAAGCCGCTACCCCGACCAC
PAM-TTAA-R	TAGCGGCTTAAGCACTGCACGCCGTAGGTC
PAM-TTCA-F	GTGCAGTGCTTCAGCCGCTACCCCGACCAC
PAM-TTCA-R	TAGCGGCTGAAGCACTGCACGCCGTAGGTC
PAM-TTCT-F	GTGCAGTGCTTCTGCCGCTACCCCGACCAC
PAM-TTCT-R	TAGCGGCAGAAGCACTGCACGCCGTAGGTC
PAM-TTCC-F	GTGCAGTGCTTCCGCCGCTACCCCGACCAC
PAM-TTCC-R	TAGCGGCGGAAGCACTGCACGCCGTAGGTC
PAM-TTCG-F	GTGCAGTGCTTCGGCCGCTACCCCGACCAC
PAM-TTCG-R	TAGCGGCCGAAGCACTGCACGCCGTAGGTC
PAM-TTGA-F	GTGCAGTGCTTGAGCCGCTACCCCGACCAC
PAM-TTGA-R	TAGCGGCTCAAGCACTGCACGCCGTAGGTC
PAM-TTGC-F	GTGCAGTGCTTGCGCCGCTACCCCGACCAC
PAM-TTGC-R	TAGCGGCGCAAGCACTGCACGCCGTAGGTC
PAM-TTGT-F	GTGCAGTGCTTGTGCCGCTACCCCGACCAC
PAM-TTGT-R	TAGCGGCACAAGCACTGCACGCCGTAGGTC
PAM-TTGG-F	GTGCAGTGCTTGGGCCGCTACCCCGACCAC
PAM-TTGG-R	TAGCGGCCCAAGCACTGCACGCCGTAGGTC
