# Supplementary material for: Structures of apo Cas12a and its complex with crRNA and DNA reveal the dynamics of ternary complex formation and target DNA cleavage
Source: PLoS Biol. 2023 Mar 14;21(3):e3002023. doi: 10.1371/journal.pbio.3002023 (PMC10013913; doi:10.1371/journal.pbio.3002023)

S1 Raw Image 1

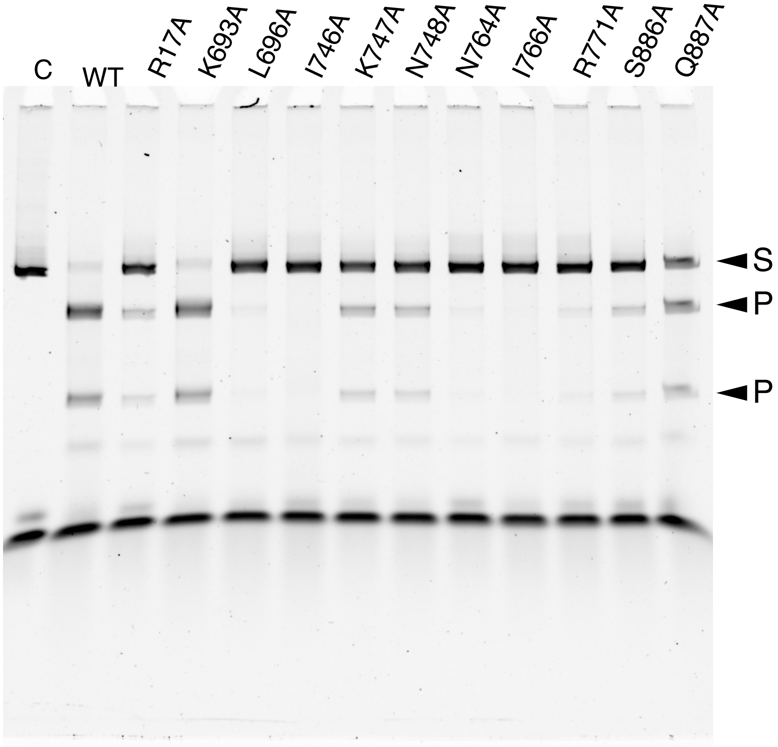

S1 Raw Image 2

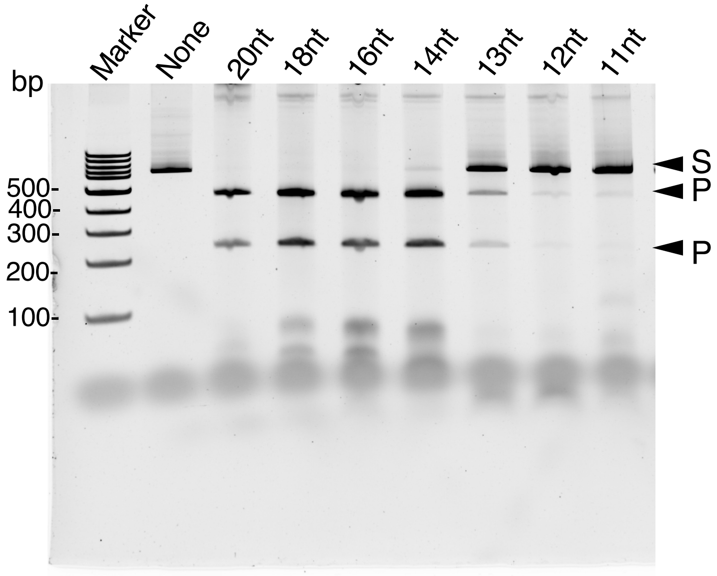

S1 Raw Image 3

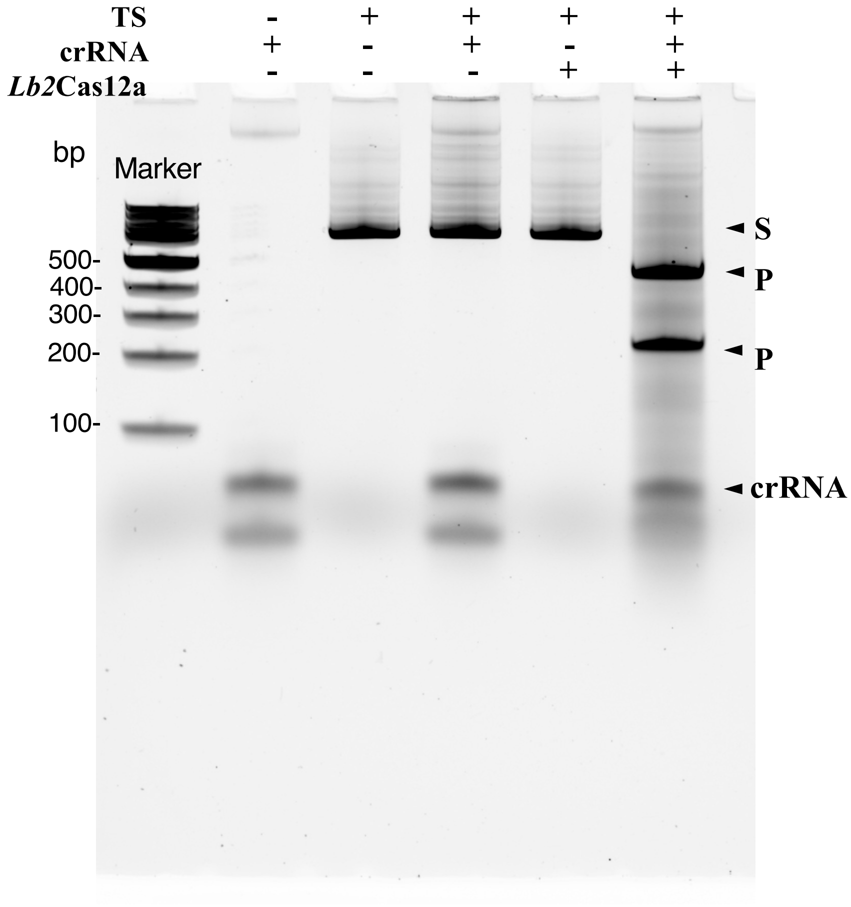

S1 Raw Image 4

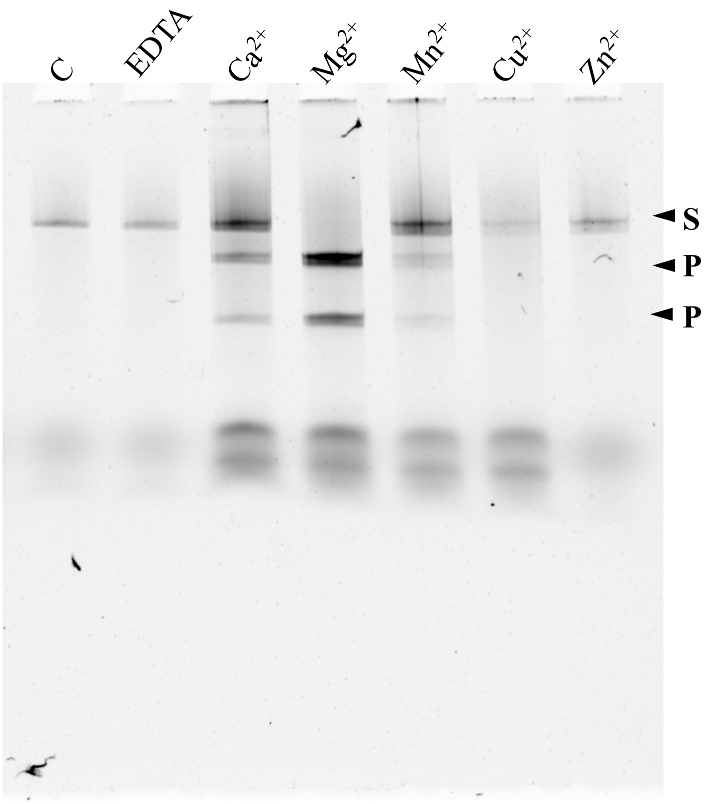

S1 Raw Image 5

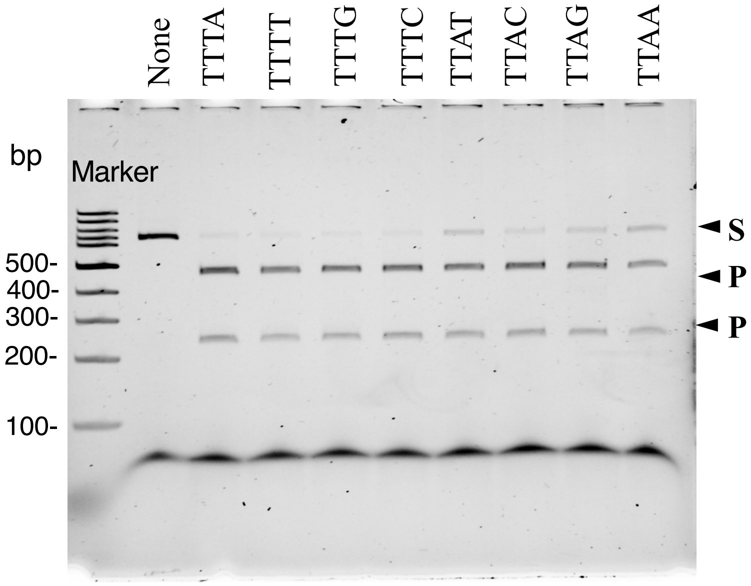

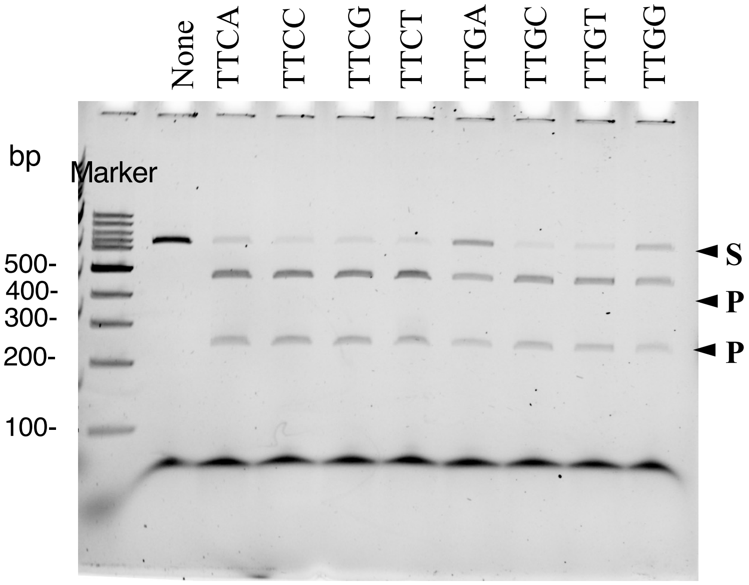

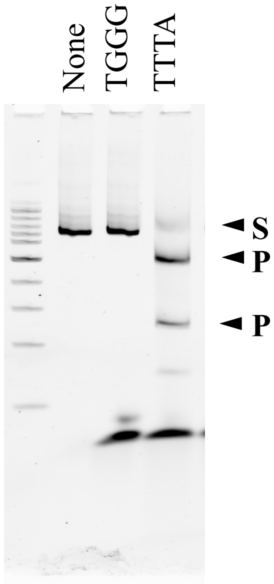

S1 Raw Image 6

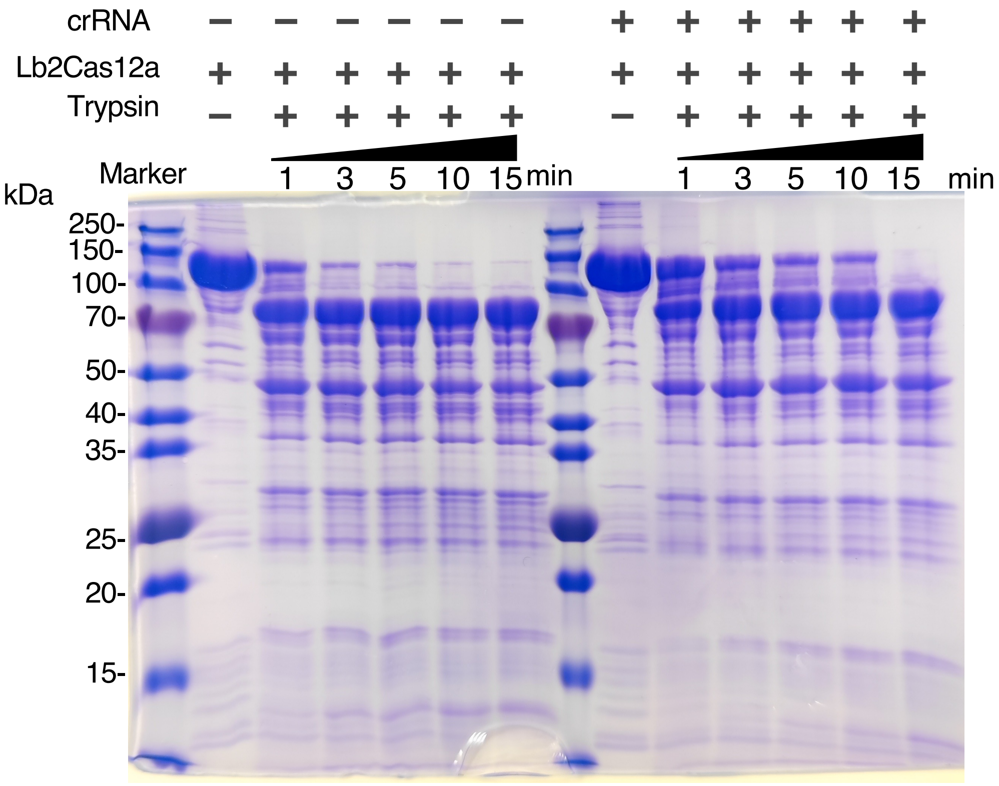

S1 Raw Image 7

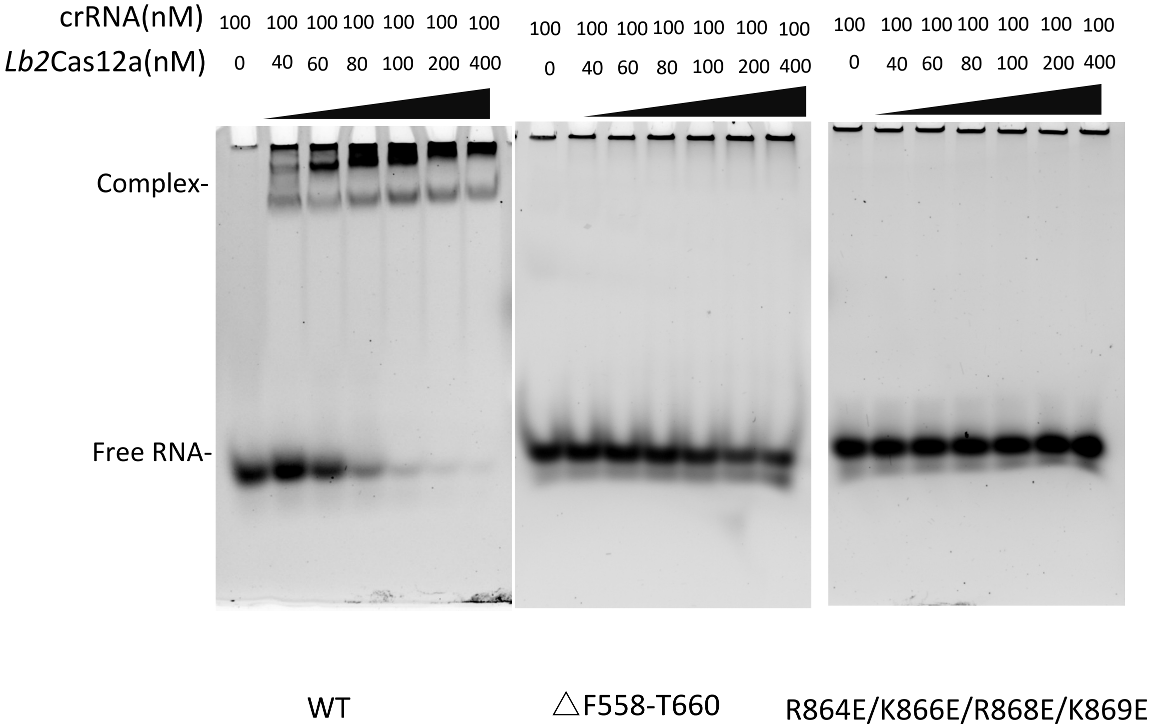

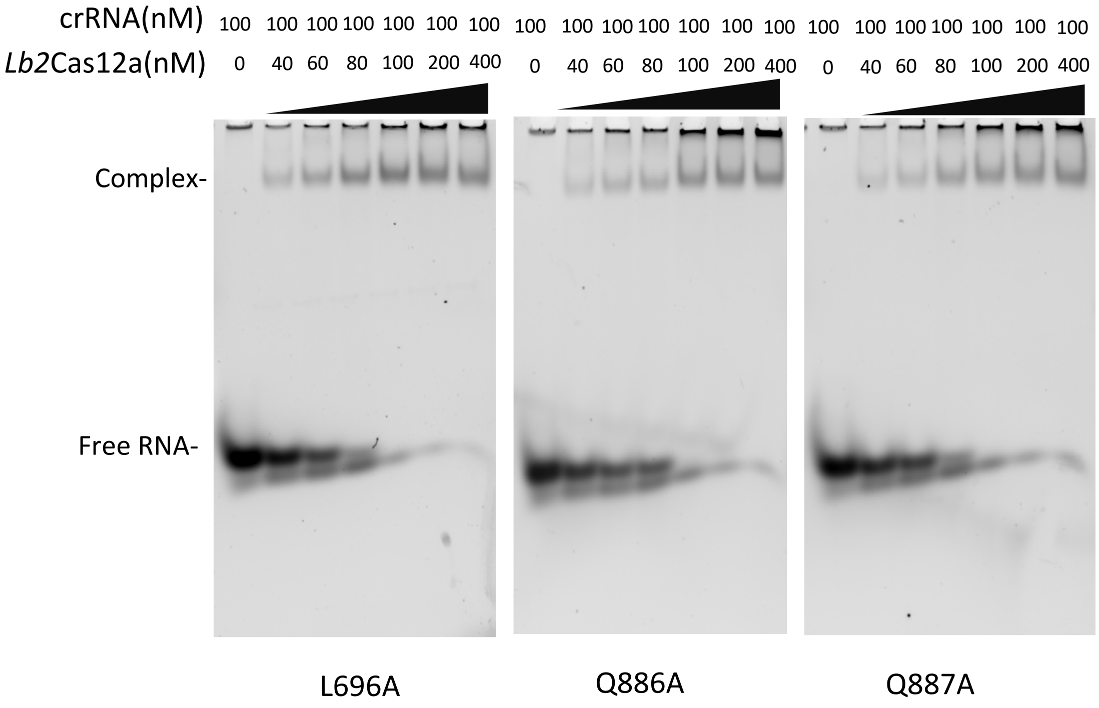

S1 Raw Image 8

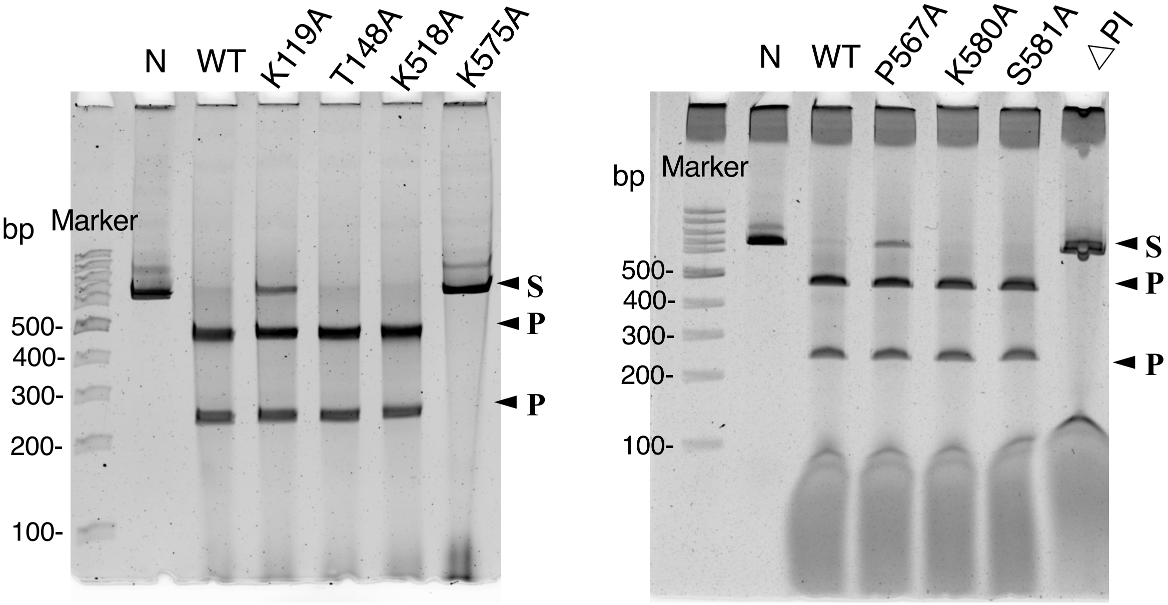

S1 Raw Image 9

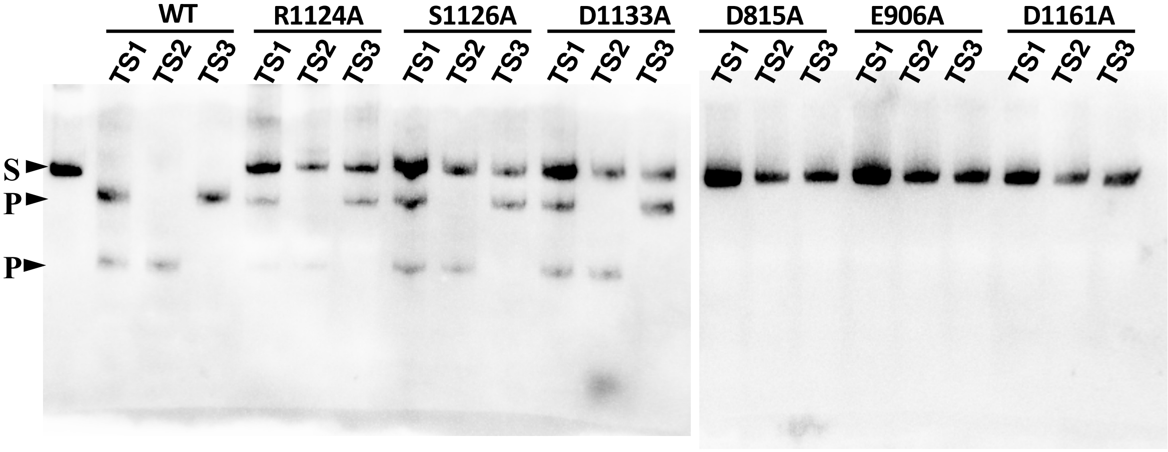

S1 Raw Image10

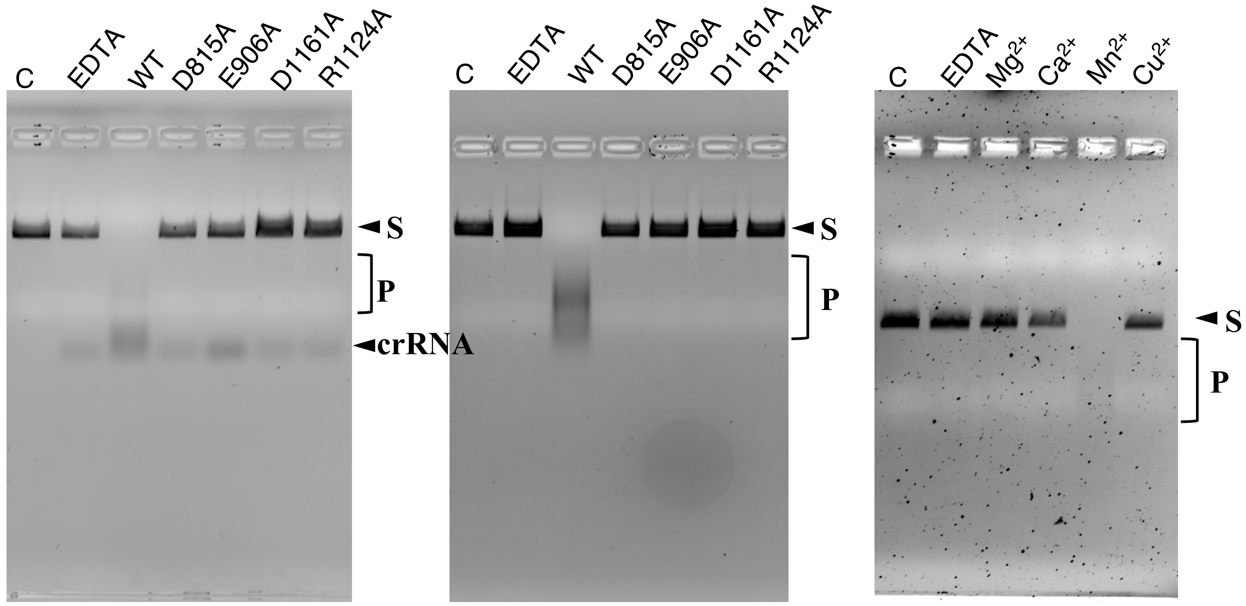

Supplement: S1 Raw Images — Image 1. Recognition of the crRNA by Lb2Cas12a. dsDNA cleavage activity analysis of Lb2Cas12a mutants in RNA recognition region. C: Control; only target dsDNA, S: Substrate, P: Cleaved product. This figure relates to the Fig 2C. Image 2. The guide strand length of crRNA required for Lb2Cas12a to achieve cleavage activity. None: only target dsDNA, S: target dsDNA substrate, P: cleavage product. This figure relates to the Fig 4C. Image 3. crRNA-dependent double-stranded DNA cleavage by Lb2Cas12a. dsDNA substrate cleavage by Lb2Cas12a with crRNA, TS: Target dsDNA; C: Control, dsDNA only; S: Substrate dsDNA; P: Cleaved products. This figure relates to the S1A Fig. Image 4. dsDNA substrate cleavage by Lb2Cas12a with different metal ion. C: Control, dsDNA only; S: Substrate dsDNA, P: Cleaved products. This figure relates to the S1C Fig. Image 5. 5′-TTNN-3′ PAM nucleotide preferences of Lb2Cas12a. None: only target dsDNA, S: Substrate dsDNA, P: Cleaved products. This figure relates to the S1D Fig. Image 6. Trypsinization of apo Lb2Cas12a and Lb2Cas12a-crRNA complex. apo Lb2Cas12a and Lb2Cas12a-crRNA complex were treated with trypsin and were resolved by SDS-PAGE. This figure relates to the S3B Fig. Image 7. EMSA of wild type and mutants of Lb2Cas12a. WT: Wild type, ΔF558 ~ T660: the truncation of Lb2Cas12a that removed PI domain. R864E/K866E/R868E/K869E: the variant that breaks the interaction between REC lobe and NUC lobe. L696A, Q886A, and Q887A: the variants that regulate crRNA into the RNA-binding pocket. This figure relates to the S8 Fig. Image 8. dsDNA cleavage activity analysis of mutants in PAM recognition region. S: target dsDNA substrate, P: cleavage product, N: negative control, dsDNA only. This figure relates to the S10C Fig. Image 9. Catalytic residues for dsDNA cleavage. C: Control, dsDNA only, S: Substrate dsDNA, P: Cleaved products. This figure relates to the S13B Fig. Image 10. ssDNA cleavage of Lb2Cas12a. Left, catalytic residues for ssDNA cleavage trigge [file pbio.3002023.s026.pdf]
